# Assessing the impacts of aminoethoxyvinylglycine and 1-methylcyclopropene on fruit drop, cracking, quality, and related transcript accumulation in ‘Ambrosia’ and ‘Fuji’ apples during on-the-tree ripening

**DOI:** 10.3389/fpls.2025.1629445

**Published:** 2025-08-22

**Authors:** Emily Johnson, Macarena Farcuh

**Affiliations:** Department of Plant Science and Landscape Architecture, University of Maryland, College Park, MD, United States

**Keywords:** plant growth regulators, fruit drop, cracking, ethylene, apples, gene expression

## Abstract

Fruit drop, cracking, and advanced ripening prior to fruit harvest can promote significant losses in important apple cultivars such as ‘Ambrosia’ and ‘Fuji’ grown in the mid-Atlantic. These losses result from environmental factors, delays in harvest due to the lack of red skin color development, and cultivar-specific characteristics, among others. Aminoethoxyvinylglycine (AVG) and 1-methylcyclopropene (1-MCP) are ethylene-inhibiting plant growth regulators (PGRs) that can alter preharvest fruit drop, cracking, maturity, and quality by impeding ethylene biosynthesis and perception, respectively. However, there is a knowledge gap on understanding the impacts of specific timings and dosages of such PGR treatments on ‘Ambrosia’ and ‘Fuji’ under mid-Atlantic environmental conditions. The objective of this work was to characterize and compare the impacts of specific rates of preharvest ethylene-inhibiting PGRs on fruit drop and cracking, ethylene production, physicochemical properties, skin color, and associated gene expression in ‘Ambrosia’ and ‘Fuji’ apples during on-the-tree ripening. Multivariate statistical methods for identifying meaningful correlations among the measured variables were applied. Our results show that both full-rate AVG (130 mg a.i. L^−1^) and 1-MCP (150 mg a.i. L^−1^) significantly reduced preharvest fruit cracking compared with control fruits in ‘Ambrosia’, but not in ‘Fuji’. Furthermore, among all treatments, full-rate AVG (130 mg a.i. L^−1^) treatment displayed the lowest ethylene production and gene expression of ethylene- related genes. 1-MCP (150 mg a.i. L^−1^) and half-rate AVG (65 mg a.i. L^−1^) treatments presented a reduction in the ethylene production and gene expression of ethylene-related genes when compared to control fruits. Regarding color, apple skin blush and anthocyanin-related gene expression were the lowest in full-rate AVG (130 mg a.i. L^−1^)-treated fruits, explaining why these fruits met the 50% red blush coverage 1 week later than all other treatments in both cultivars. Correlations amongst the assessed features were also identified. These results suggest a cultivar-specific effectiveness under mid-Atlantic conditions and specifies a framework for the use of ethylene-inhibiting PGRs under mid-Atlantic environmental conditions.

## 1 Introduction

Preharvest fruit drop and cracking due to abscission from the tree and fruit fissures prior to the optimal maturity increases yield losses (Arseneault and Cline, 2016; Li et al., 2021). The strategy of harvesting apple fruit before the optimal maturity to reduce fruit drop and cracking negatively impacts red skin color development while reducing fruit quality and marketability (Kvikliene et al., 2011; Anbesse Girma et al., 2022). Therefore, finding new horticultural practices that allow harvest at optimal maturity while enhancing red skin coloration, but without promoting fruit drop or cracking, is a critical challenge for commercially important cultivars such as ‘Ambrosia’ and ‘Fuji’ grown under mid-Atlantic conditions.

‘Ambrosia’ is an emerging mid-season cultivar in the USA, primarily restricted to Canada until 2017 (Warner, 2025). This cultivar presents a challenge to the industry, as it possesses a narrow harvest period that is hard to navigate when managing cultivars with overlapping harvest windows and seems to present susceptibility to fruit drop, yet predisposition to cracking is generally unknown (Mennell, 2002; Crassweller et al., 2007; Toivonen, 2015). ‘Fuji’ is a late-season cultivar, favored by growers and consumers and maintaining popularity in the top five U.S. cultivars (Peng and Fu, 2023). Previous work has reported ‘Fuji’ to display a high sensitivity to preharvest fruit cracking, contributing to yield losses, although susceptibility to fruit drop has been inconsistent under different environmental conditions where the fruits are grown (Kasai et al., 2008; Li et al., 2010). To our knowledge, understanding of the predisposition of ‘Ambrosia’ and ‘Fuji’ cultivars to fruit drop and cracking under mid-Atlantic conditions is limited.

Preharvest fruit drop, cracking, and fruit quality are known to be highly regulated by ethylene, a key plant hormone responsible for fruit ripening along with a host of other physiological processes (Bleecker and Schaller, 1996; Santos et al., 2023). Apples are climacteric fruits and thus exhibit a surge in ethylene production concurrent with an increase in respiration throughout ripening (Giovannoni, 2004; Chen et al., 2018). As a positive regulator of abscission, ethylene induces intracellular tissue and cell wall degradation in the abscission zone of the fruit pedicel and therefore increases preharvest fruit drop as the ripening proceeds throughout the season (Estornell et al., 2013; Arseneault and Cline, 2016; Miah and Farcuh, 2024a). Further, ethylene seems to be implicated in the regulation of fruit cracking through indirect mechanisms, which increases with advanced fruit maturity and ripening and is additionally associated with cell wall regulation-related genes that are ethylene-responsive (Khadivi-Khub, 2015; La Spada et al., 2024). The ethylene pathway has been widely studied, and its components are well documented (Klee, 2004; Binder, 2020; Liu et al., 2020; Cocetta and Natalini, 2022; Kader, 2022). The ethylene pathway is composed of ethylene biosynthesis, involving the enzymatic catalysis of *S*-adenosyl-l-methionine (SAM) to 1-aminocyclopropane-1-carboxylate (ACC) by the enzyme ACS and to ethylene by the enzyme 1-aminocyclopropane-1-carboxylate oxidase (ACO) (Adams and Yang, 1979; Bleecker and Schaller, 1996; Farcuh et al., 2018; Wang and Faust, 2019); ethylene perception, comprising copper-binding membrane-associated receptors [ethylene response sensor (ERS) and ethylene receptor type (ETR)] and a negative regulator of ethylene response [constitutive triple response 1 (*MdCTR1*), a Ref-like serine/threonine-protein kinase] (Lee et al., 1998; Dal Cin et al., 2005, 2006; Wiersma et al., 2007; Yuan and Li, 2008; Busatto et al., 2017; Dolgikh et al., 2019; Bleecker, 1999); and lastly, ethylene signaling, activated by several transcription factors, including the ethylene response factors (ERFs) (Li et al., 2016).

Ethylene-inhibiting plant growth regulators (PGRs) that alter the ethylene pathway are commonly utilized to control preharvest fruit drop and cracking in apples. Two such PGRs are aminoethoxyvinylglycine (AVG), which impedes ACS activity in ethylene biosynthesis (Brasil and Siddiqui, 2018; Liu et al., 2022; Miah and Farcuh, 2024c), and 1-methylcyclopropene (1-MCP), which binds to and saturates receptor sites involved in ethylene perception (Doerflinger et al., 2019). The rate and timing of AVG and 1-MCP have been reported to impact their effectiveness on apple fruit drop and cracking, while location, yearly variation, and the cultivar type also contribute to the efficacy of these ethylene-inhibiting PGRs (Argenta et al., 2018a). Although previous research has shown that AVG and 1-MCP both exhibit a reduction in preharvest fruit drop in ‘Delicious’ (Yuan and Li, 2008; Malladi et al., 2023), ‘Honeycrisp’ (Arseneault and Cline, 2017; Johnson and Farcuh, 2024; Miah and Farcuh, 2024a), ‘Gala’ (Layne et al., 2002; Argenta et al., 2018a; Liu et al., 2022), ‘McIntosh’ (Schupp and Greene, 2004; Greene, 2005), and ‘Golden Supreme’ (Yuan and Carbaugh, 2007) apples, while additionally demonstrating reduced cracking in ‘Gala’ (Lee et al., 2016; Liu et al., 2022), different dosages and timings of application were used in each study, depending on cultivar as well as growing environment, hence indicating that generalizations on recommendations cannot be made and that specific studies are necessary for different cultivars grown under different environmental conditions to define the most efficient application dosages and timing. To date, we are not aware of any previous work that has investigated the impacts of specific dosages and timings of these ethylene-inhibiting PGRs on ‘Ambrosia’ and ‘Fuji’ grown under mid-Atlantic conditions. This work is of critical need in the region, directly boosting fruit quality, production, and profitability.

Ethylene plays a pivotal role in modulating fruit ripening and influencing key quality attributes, including texture, flavor, and color, which are crucial for consumer preference (Giovannoni, 2004; Kumar et al., 2014; Miah et al., 2023). Fruits treated with ethylene-inhibiting PGRs, particularly AVG and 1-MCP, have been shown to maintain apple flesh firmness and decrease starch hydrolysis to sugars in ‘Gala’ (Argenta et al., 2018b), ‘Cripps Pink’ (do Amarante et al., 2022), ‘McIntosh’ (Doerflinger et al., 2019), and ‘Red Delicious’ apples (Boyacı, 2022). However, changes in fruit titratable acidity and soluble solids content have exhibited incongruities, with no significant changes after ethylene-inhibiting PGR treatments (McArtney et al., 2008; do Amarante et al., 2022) in some cultivars and higher acidity and lower soluble solids in others (Liu et al., 2022; Malladi et al., 2023; Doerflinger et al., 2024; Miah and Farcuh, 2024a). Moreover, different ethylene-inhibiting PGR treatments have also shown discrepancies in the development of apple red skin coloration, proving problematic for the stringent requirements for U.S. standards of marketability (USDA, 2019). Preharvest AVG-treated fruits have previously exhibited delays to red coloration in ‘Gala’ (Liu et al., 2022) and ‘Cripps Pink’ (Whale et al., 2008), yet did not display significant changes to coloring in ‘Delicious’ (Yuan and Li, 2008), while preharvest 1-MCP-treated apples showed red skin color development delays in ‘Anna’ (Farag et al., 2015) and ‘Delicious’ (Yuan and Li, 2008) and no differences in red skin coloration in ‘Scarletspur Delicious’ (Elfving et al., 2007). These inconsistencies have been shown to result from the alteration in the biosynthesis of anthocyanins, the primary pigment responsible for apple red skin coloration, which can directly impact apple fruit marketability (Liu et al., 2021; Fanyuk et al., 2022). Anthocyanin biosynthesis occurs via the phenylpropanoid metabolic pathway, beginning with the precursor phenylalanine, derived from the shikimate pathway and enzymatically converted to anthocyanin in the flavonoid pathway (Whale et al., 2008; Fanyuk et al., 2022). The enzymes included in the conversion are phenylalanine ammonia-lyase (PAL), chalcone synthase (CHS), chalcone isomerase (CHI), flavanone 3-hydroxylase (F3H), dihydroflavonol 4-reductase (DFR), leucoanthocyanidin dioxygenase (LDOX), and UDP-glucose-flavonoid 3-*O*-glucosyltransferase (UFGT), as well as *MdMYB10*, a regulatory transcription factor exhibiting a rise in parallel with anthocyanin content (Whale et al., 2008; Feng et al., 2013; Ma et al., 2021; Li et al., 2022; Shi et al., 2022; Sunil and Shetty, 2022; Wang et al., 2022). Although ethylene has been shown to play a major role in upregulating anthocyanin biosynthesis-related genes (Elfving et al., 2007; Onik et al., 2018; Zhang et al., 2018; Farcuh et al., 2022), notably, as described above, different ethylene-inhibiting PGR treatments have displayed inconsistent results in red skin coloration when applied to different apple cultivars. This may indicate that other factors, in addition to ethylene, such as cultivar, environmental conditions, specific ethylene PGR applied, and timings and dosages of each PGR, may also play key roles, which needs further investigation. Particularly for ‘Ambrosia’ and ‘Fuji’ apples grown in the mid-Atlantic, information is lacking on the impact of different ethylene-inhibiting PGRs on fruit red skin coloration.

Based on the available literature, studies examining the influence of ethylene-inhibiting PGR treatments on preharvest fruit drop, cracking, fruit maturity, and quality characteristics, as well as associated ethylene and anthocyanin transcript accumulation, are lacking, particularly in important cultivars for the mid-Atlantic region, such as ‘Ambrosia’ and ‘Fuji’. Accordingly, the objective of this study was to depict and contrast the effects of specific rates of preharvest ethylene-inhibiting PGRs (AVG and 1-MCP) on fruit drop and cracking, ethylene production, physicochemical characteristics, skin color, and expression of genes involved in ethylene biosynthesis and its perception, as well as genes responsible for anthocyanin biosynthesis in ‘Ambrosia’ and ‘Fuji’ apples during preharvest ripening. Multivariate statistical methods were applied to identify meaningful correlations among the measured variables.

## 2 Materials and methods

### 2.1 Plant materials and treatments

#### 2.1.1 Experiment 1: ‘Ambrosia’

A field trial was conducted over two consecutive years using a 6-year-old ‘Ambrosia’ apple orchard (*Malus domestica* ‘Ambrosia’/M9) located in Aspers, PA. The trees were trained to a modified central leader system on a trellis and planted at a spacing of 1 × 4 m. A randomized complete block design was implemented, consisting of four treatments, each replicated four times with 40 trees per replicate. Four treatments were applied: AVG at 130 and 65 mg a.i. L^−1^ (ReTain, Valent BioSciences Corporation, Libertyville, IL, USA), 1-MCP at 150 mg a.i. L^−1^ MCP (Harvista 1.3 SC, AgroFresh, Philadelphia, PA, USA), and an untreated control. The full-rate (130 mg a.i. L^−1^) of AVG was applied 4 weeks prior to the expected commercial harvest date, and the half-rate (65 mg a.i. L^−1^) was applied 1 week prior to the expected commercial harvest date. Rates and application timings were based on manufacturer recommendations. ‘Ambrosia’ is suggested to be a sensitive cultivar that, in addition to the full-rate, may respond to lower application rates closer to the anticipated harvest (Valent BioSciences, 2022). 1-MCP was applied when the starch pattern index reached 3, based on manufacturer recommendations, using an AgroFresh formulation tank, injection pump, and calibration tube attached to a sprayer. AVG and 1-MCP treatments were combined with 1.0 mL L^−1^ Silwet-77 organosilicone surfactant and applied with a pressurized orchard sprayer.

Maturity indices were assessed throughout the season [i.e., surface and background color, skin blush, flesh firmness, starch pattern index (SPI), soluble solids content (SSC), and titratable acidity (TA)] to determine the anticipated commercial harvest date following prior methodology (Miah and Farcuh, 2024c) and using control fruits as the reference. Three evaluation timepoints during on-the-tree ripening were selected: 1 week before commercial harvest (1WBCH), commercial harvest (CH), and 1 week after commercial harvest (CH + 1W). At each timepoint, eight fruits per replicate were harvested for each treatment and promptly brought to the laboratory. Per replication, four fruits were used for measuring ethylene production rate and rinsed, the skin was removed (skin tissue), and the remaining flesh was cut (flesh tissue). The remaining fruits were evaluated for physicochemical parameters.

#### 2.1.2 Experiment 2: Fuji

A field trial was conducted over two consecutive years using a 15-year-old ‘Fuji’ apple orchard (*M. domestica* ‘Fuji’/M9) located in Aspers, PA. The trees were trained to a tall spindle and planted at a spacing of 1 × 4 m. A randomized complete block design was implemented, consisting of three treatments, each replicated four times with 40 trees per replicate. The three treatments included full-rate AVG at 130 mg a.i. L^−1^, 1-MCP at 150 mg a.i. L^−1^ MCP, and an untreated control, applied as described above for Experiment 1.

Fruits were harvested at three different timepoints during the tree ripening—1WBCH, CH, and CH + 1W—following what was described above for Experiment 1.

### 2.2 Determination of preharvest fruit drop and cracking

For both experiments, independently, preharvest fruit drop was assessed as previously described (Johnson and Farcuh, 2024; Miah and Farcuh, 2024b) for three treatments: full-rate AVG at 130 mg a.i. L^−1^, 1-MCP at 150 mg a.i. L^−1^ MCP, and the control. For each replication, preharvest fruit drop was calculated from five pre-selected limbs labeled 2 weeks before CH, containing 20 fruits each on alternate sides of different trees throughout the block. Fruit drop was assessed on each harvest timepoint, i.e., starting at 1WBCH and ending at CH + 1W. The number of fruits on each limb was counted at each evaluation timepoint, and fruit drop was calculated as a percentage of the original fruit count on that limb. The percentage of cracked fruits was additionally determined as a count of cracked fruits per limb at each timepoint (Byers, 1997).

### 2.3 Ethylene production assessment

For Experiment 1 (‘Ambrosia’), ethylene production rate (μL C_2_H_4_ kg^−1^ h^−1^) was measured via a static system as previously described (Tong et al., 2003; Kim et al., 2015a; Farcuh and Hopfer, 2023). ‘Ambrosia’ fruits were incubated in a hermetic 1-L jar equipped with rubber stoppers for 1 hour at 20°C. One milliliter of headspace gas was drawn from each jar with a syringe. For Experiment 2 (‘Fuji’), internal ethylene concentration (IEC) was determined from 1-mL samples of internal gas pulled from the core cavity, as portrayed in previous research (Miah et al., 2023; Miah and Farcuh, 2024a). The obtained gas samples were injected into a gas chromatograph (GC-2014C, Shimadzu Co., Kyoto, Japan) equipped with an activated alumina column and a Flame ionization detector (FID) as previously described (Johnson and Farcuh, 2024).

### 2.4 Fruit skin color and physicochemical analyses

Evaluation of blush percentage, skin color, index of absorbance difference (I_AD_), flesh firmness, SPI, SSC, and TA was conducted as reported earlier (Miah et al., 2023; Miah and Farcuh, 2024c). Fruit blush percentage was visually estimated to the nearest 5% on each apple. Surface and background color were evaluated using a colorimeter (Konica Minolta CR400 Chroma Meter, Konica Minolta Sensing, Inc., Osaka, Japan), and hue angle (hue °) was estimated as previously explained (Infante et al., 2008). The I_AD_ was measured utilizing a Delta Absorbance (DA) Meter (TR Turoni, Forli, Italy) with measurements taken from three distinct fruit surface points and averaged (Ziosi et al., 2008). Flesh firmness was assessed on the two opposing fruit sides, with approximately 2-mm thickness peeled from each side using a TA.XT Plus Connect texture analyzer (Texture Technologies Corp., Scarsdale, NY, USA) equipped with an 11.1-mm-diameter probe, a 50-kg load cell, and the Exponent TE32 software (v6.0, Texture Technologies Corp., Scarsdale, NY, USA) operated at 8 mm with a speed of 8 mm s^−1^. For SPI measurements, each fruit was halved transversally and rated between 1 (100% stained starch) and 8 (0% stained starch) using the Cornell generic chart (Blanpied and Silsby, 1992). SSC and malic acid were measured using a hand-held digital refractometer (Atago, Tokyo, Japan) and an automatic titrator (855 Robotic Titrosampler; Metrohm, Riverview, FL, USA), respectively (Farcuh et al., 2020, 2018; Miah and Farcuh, 2024b).

### 2.5 Total RNA extraction and real-time quantitative polymerase chain reaction

The isolation of RNA took place from skin and flesh tissue from each replication at each evaluation timepoint using a modified cetyltrimethylammonium bromide (CTAB)/NaCl method (Chang et al., 1993) as explained earlier (Kim et al., 2015b; Farcuh et al., 2018; Miah and Farcuh, 2024a). The synthesis of first-strand complementary DNA, primer design, and quantitative real-time polymerase chain reaction occurred as reported earlier (Kim et al., 2015b), with primer sequences shown in **Supplementary Table 1**. Relative gene expression examination was performed employing the Comparative Cycle Threshold Method (Livak and Schmittgen, 2001) with the reference gene actin (*MdACT*).

### 2.6 Statistical analyses

A factorial experiment under a randomized complete block design was analyzed in RStudio using linear mixed models with treatment and evaluation timepoints as fixed factors and replications as the random factor (R ver 2024.04.2 + 764). Normality was confirmed from the residuals, and Tukey’s honestly significant difference (HSD) at 5% significance level was used to compare the treatments when the model was statistically significant.

Pearson’s correlation coefficients were calculated for each pairwise combination of factors using mean-centered data in the software package JMP (ver 15.2, SAS Institute, Cary, NC, USA). A “biplot” graph was used to visualize the principal component analysis (PCA) for associations among the assessed treatments and evaluation timepoints, as well as analyzed features (fruit drop, fruit cracking, ethylene production, skin color, physicochemical parameters, and gene expression values), with the number of principal components determined using the scree test.

## 3 Results

### 3.1 Experiment 1: effects of AVG and 1-MCP on ‘Ambrosia’ fruit drop, cracking, ethylene production, physicochemical parameters, skin color, and gene expression during on-the-tree ripening

#### 3.1.1 Fruit drop and cracking in ‘Ambrosia’

For preharvest fruit drop and cracking in ‘Ambrosia’ fruits, only three out of the four treatments were evaluated throughout on-the-tree ripening at the three harvest timepoints (full-rate AVG, 1-MCP, and control) (**Table 1**). In the case of fruit drop, a trend of increased fruit drop was observed for most treatments throughout on-the-tree ripening over both years. Although differences were not statistically significant between treatments in preharvest fruit drop, at CH + 1W, AVG treatments exhibited a 50% reduction in fruit drop compared to the control in 2023, while in 2024, both AVG and 1-MCP treatments displayed 1.5-fold lower fruit drop percentages than control fruits.

**Table 1:** Effect of AVG and 1-MCP on ‘Ambrosia’ preharvest fruit drop and cracking evaluated during on-the-tree ripening in Aspers, PA, during 2023 and 2024 production seasons.

Year	Treatment	Preharvest fruit drop (%)			Preharvest fruit cracking (%)		
		1WBCH	CH	CH + 1W	1WBCH	CH	CH + 1W
2023							
	AVG	1.15 ± 0.44 c	3.71 ± 1.01 bc	4.93 ± 1.48 abc	0 ± 0.00 c	0 ± 0.00 c	3.72 ± 1.23 b
	1-MCP	3.3 ± 0.86 c	7.25 ± 0.60 ab	8.38 ± 0.22 a	0 ± 0.00 c	0.54 ± 0.32 c	4.76 ± 0.98 ab
	Control	3.44 ± 1.22 bc	6.37 ± 2.28 abc	10.9 ± 1.87 ab	0.25 ± 0.25 c	0.25 ± 0.25 c	7.29 ± 0.39 a
2024							
	AVG	0.95 ± 0.38 b	2.11 ± 0.54 ab	4.44 ± 1.11 ab	0 ± 0.00 b	0.48 ± 0.28 b	1.39 ± 0.45 b
	1-MCP	1.09 ± 0.42 b	2.78 ± 1.18 ab	4.52 ± 1.36 ab	0 ± 0.00 b	0.25 ± 0.25 b	1.19 ± 0.27 b
	Control	3.74 ± 0.85 ab	3.04 ± 0.43 ab	7.2 ± 2.14 a	0 ± 0.00 b	0.75 ± 0.48 b	3.53 ± 0.64 a

Apples were harvested at 1 week before commercial harvest (1WBCH), commercial harvest (CH), and 1 week after CH (CH + 1W). Values are means ± standard error. Distinct letters represent statistically significant differences (p ≤ 0.05) according to Tukey’s HSD test.

AVG, aminoethoxyvinylglycine; 1-MCP, 1-methylcyclopropene.

Regarding fruit cracking, values increased remarkably across ripening timepoints from 1WBCH to CH + 1W for all treatments in 2023 and control fruits in 2024. In 2023, AVG-treated fruits exhibited a significant twofold reduction in fruit cracking compared to the control, while in 2024, both AVG- and 1-MCP-treated fruits displayed a significant 2.5-fold decrease in values compared to the control.

#### 3.1.2 Ethylene production and physicochemical parameters in ‘Ambrosia’

**Figure 1:**
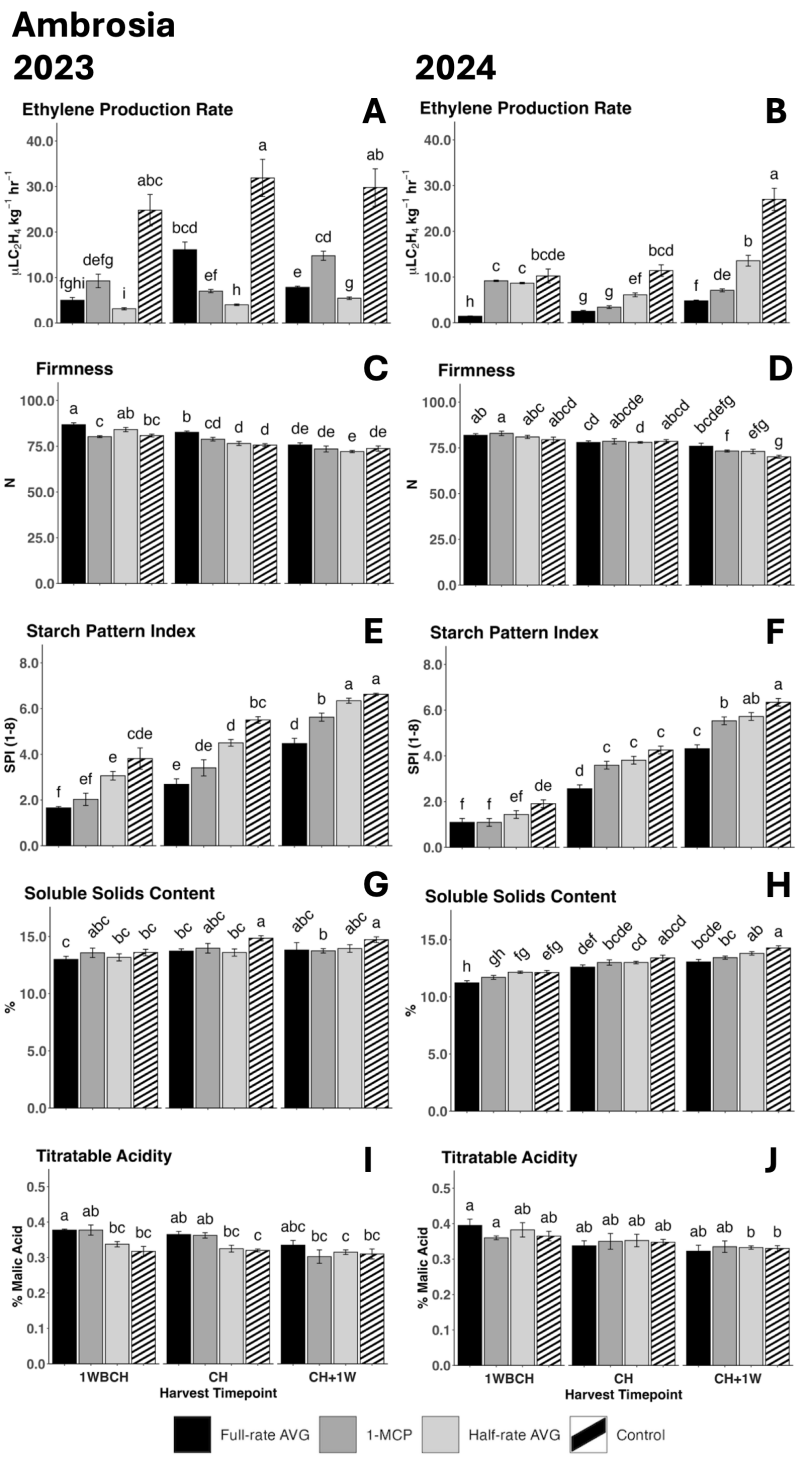
Effect of full-rate AVG, half-rate AVG, and 1-MCP on ‘Ambrosia’ ethylene production rate and physicochemical properties during on-the-tree ripening in Aspers, PA, in **(A, C, F, H, J)** 2023 and **(B, E, G, I, K)** 2024. **(A, B)** Ethylene production rate, **(C, D)** flesh firmness, **(E, F)** starch content (starch pattern index), **(G, H)** soluble solids content, and **(I, J)** titratable acidity (malic acid). Apple evaluations were conducted at 1 week before commercial harvest (1WBCH), commercial harvest (CH), and 1 week after commercial harvest (CH + 1W). Values are means ± standard error. Distinct letters represent statistically significant differences (p ≤ 0.05) according to Tukey’s HSD test. AVG, aminoethoxyvinylglycine; 1-MCP, 1-methylcyclopropene.

Ethylene production rate displayed a significant increase from 1WBCH to CH + 1W for full-rate and half-rate AVG during the tree ripening in 2023 and for every treatment in 2024, presenting the highest values at CH + 1W for each treatment when compared across the assessed timepoints (**Figures 1A, B**). During both assayed years, control fruits showed the statistically highest ethylene production rates as compared to all other treatments during most timepoints. In 2023, half-rate AVG-treated fruits showed the least ethylene production rates at all assessed timepoints, followed by full-rate AVG-treated fruits, then 1-MCP-treated fruits, and lastly by control fruits with the highest values (**Figure 1A**). In 2024, full-rate AVG-treated fruits displayed the least ethylene production rate at 1WBCH as well as CH + 1W, and at the latter, it was followed by 1-MCP-treated fruits, then by half-rate AVG-treated fruits, and finally by control fruits, exhibiting the highest values (**Figure 1B**).

Flesh firmness was significantly reduced from 1WBCH to CH + 1W for all treatments throughout the three assayed timepoints, consistently in both years (**Figures 1C, D**). In 2023, full-rate AVG-treated fruits exhibited significantly higher firmness values than 1-MCP-treated and control fruits at 1WBCH than all other treatments at CH (**Figure 1C**). In 2024, 1-MCP-treated fruits exhibited statistically higher firmness values than control fruits at CH + 1W (**Figure 1D**).

Starch pattern index values steadily increased in both years for all treatments from 1WBCH to CH + 1W, indicative of increased starch disappearance throughout ripening (**Figures 1E, F**). In 2023, SPI values were the lowest for full-rate AVG-treated fruits at 1WBCH and CH, followed by 1-MCP-treated fruits, half-rate AVG-treated fruits, and lastly the control fruits, with the highest exhibited values (**Figure 1E**). In 2024, full-rate AVG- and 1-MCP-treated fruits presented SPI values that were significantly lower than those of control fruits at 1WBCH, while full-rate AVG-treated fruits displayed the statistically lowest SPI values at CH (**Figure 1F**). Finally, at CH + 1W, full-rate AVG-treated fruits presented the most reduced SPI values in both years among all treatments; in 2024, 1-MCP- and half-rate AVG-treated fruits exhibited intermediate SPI values compared with full-rate AVG-treated and control fruits (**Figures 1E, F**).

SSC significantly increased from 1WBCH to CH+ 1W throughout the three assessed on-the-tree ripening timepoints in all treatments during 2024 (**Figure 1H**), yet not in 2023 (**Figure 1G**). In this same year, full-rate AVG-treated fruits displayed lower SSC values than half-rate AVG-treated and control fruits at 1WBCH, while at CH + 1W, both full-rate AVG- and 1-MCP-treated fruits exhibited statistically lower SSC values than control fruits.

TA values displayed a decreasing trend as on-the-tree ripening progressed from 1WBCH to CH + 1W for each treatment in 2023 and 2024 (**Figures 1I, J**). In 2023, full-rate AVG- and 1-MCP-treated fruits displayed statistically significantly higher TA values than control fruits at CH (**Figure 1I**).

#### 3.1.3 Skin coloration in ‘Ambrosia’

**Figure 2:**
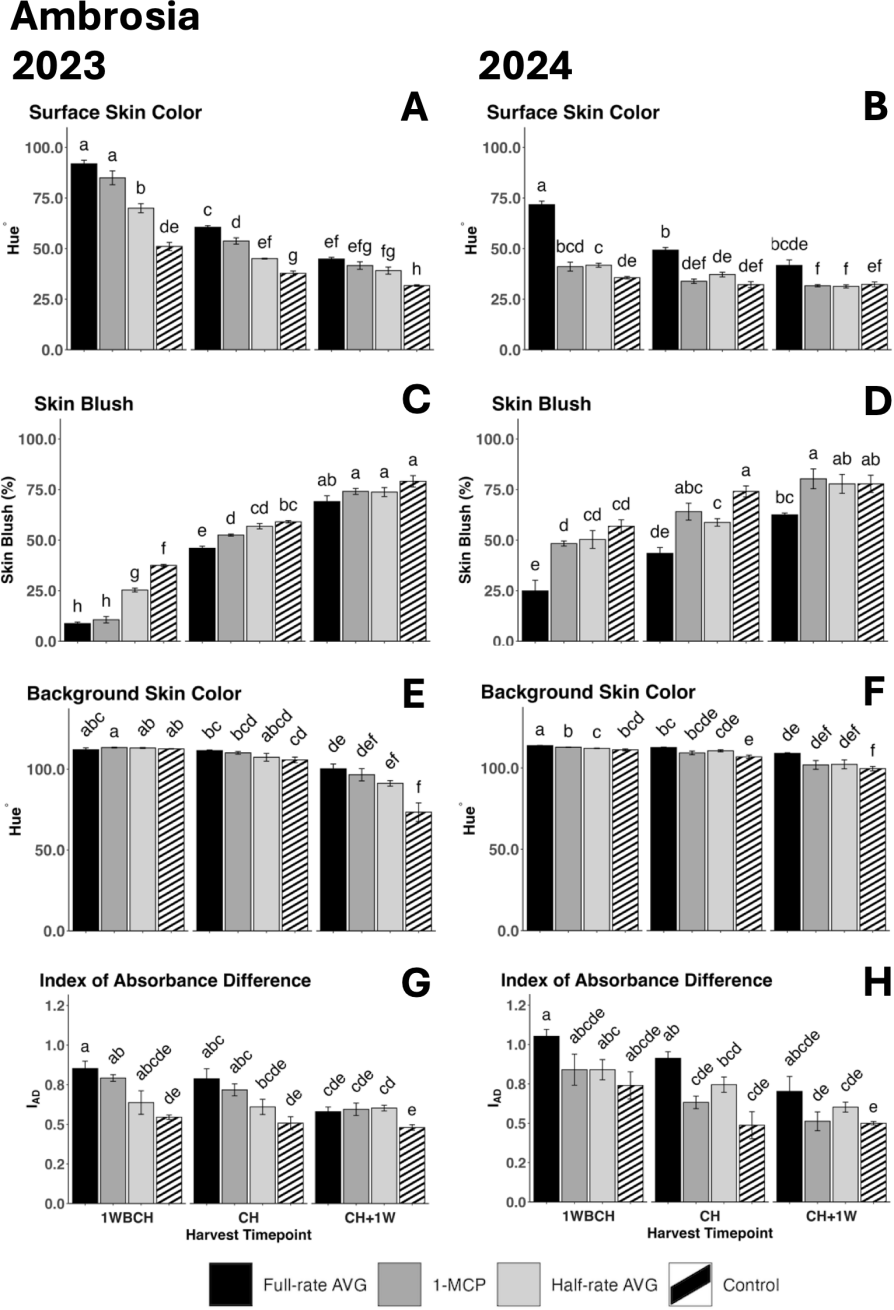
Effect of full-rate AVG, half-rate AVG, and 1-MCP on ‘Ambrosia’ fruit color during on-the-tree ripening in Aspers, PA, in **(A, C, E, G)** 2023 and **(B, D, F, H)** 2024. **(A, B)** Surface skin color, **(C, D)** skin blush, **(E, F)** background skin color, and **(G, H)** index of absorbance difference. Apple evaluations were conducted at 1 week before commercial harvest (1WBCH), commercial harvest (CH), and 1 week after commercial harvest (CH + 1W). Values are means ± standard error. Distinct letters represent statistically significant differences (p ≤ 0.05) according to Tukey’s HSD test. AVG, aminoethoxyvinylglycine; 1-MCP, 1-methylcyclopropene.

A significant decrease in surface skin hue values from 1WBCH to CH + 1W was observed in both years, revealing an increased red skin coloration (**Figures 2A, B**). In 2023, at 1WBCH, full-rate AVG- and 1-MCP-treated fruits showed the highest surface skin values, followed by half-rate AVG-treated fruits, and finally by control fruits, while at CH, full-rate AVG-treated fruits significantly differentiated with the highest values from all other treatments (**Figure 2A**). Control fruits exhibited the statistically lowest values at CH + 1W (**Figure 2A**). In 2024, full-rate AVG-treated fruits displayed the highest surface skin hue values at 1WBCH and CH (**Figure 2B**). Correspondingly, skin blush percentage increased significantly, for all treatments, as fruits ripened on the tree (**Figures 2C, D**). Skin blush percentage was statistically the lowest for full-rate AVG- and 1-MCP-treated fruits, followed by half-rate AVG-treated fruits, and finally by control fruits, which exhibited the highest blush values at 1WBCH in 2023 (**Figure 2C**). At CH in 2023 and 1WBCH and CH in 2024, full-rate AVG treatment displayed the least blush values compared with all other treatments. Notably, 1-MCP-treated, half-rate AVG-treated, and control fruits reached a blush percentage >50% at CH, while full-rate AVG-treated fruits achieved this 1 week later at CH + 1W in both years (**Figures 2C, D**).

A significant reduction in background skin hue values was observed in both years for every treatment from 1WBCH to CH + 1W, revealing a green to yellow color conversion (**Figures 2E, F**). Furthermore, at CH + 1W, control fruits displayed the lowest background skin hue angle values, statistically differing from full-rate AVG-treated fruits in both years (**Figures 2E, F**). Similarly, I_AD_ values were significantly lower in control fruits than in full-rate AVG-treated fruits at 1WBCH and CH in 2023 and at CH in 2024, while the rest of the treatments displayed intermediate values (**Figures 2G, H**).

#### 3.1.4 Expression of ethylene biosynthesis and perception genes in ‘Ambrosia’

**Figure 3:**
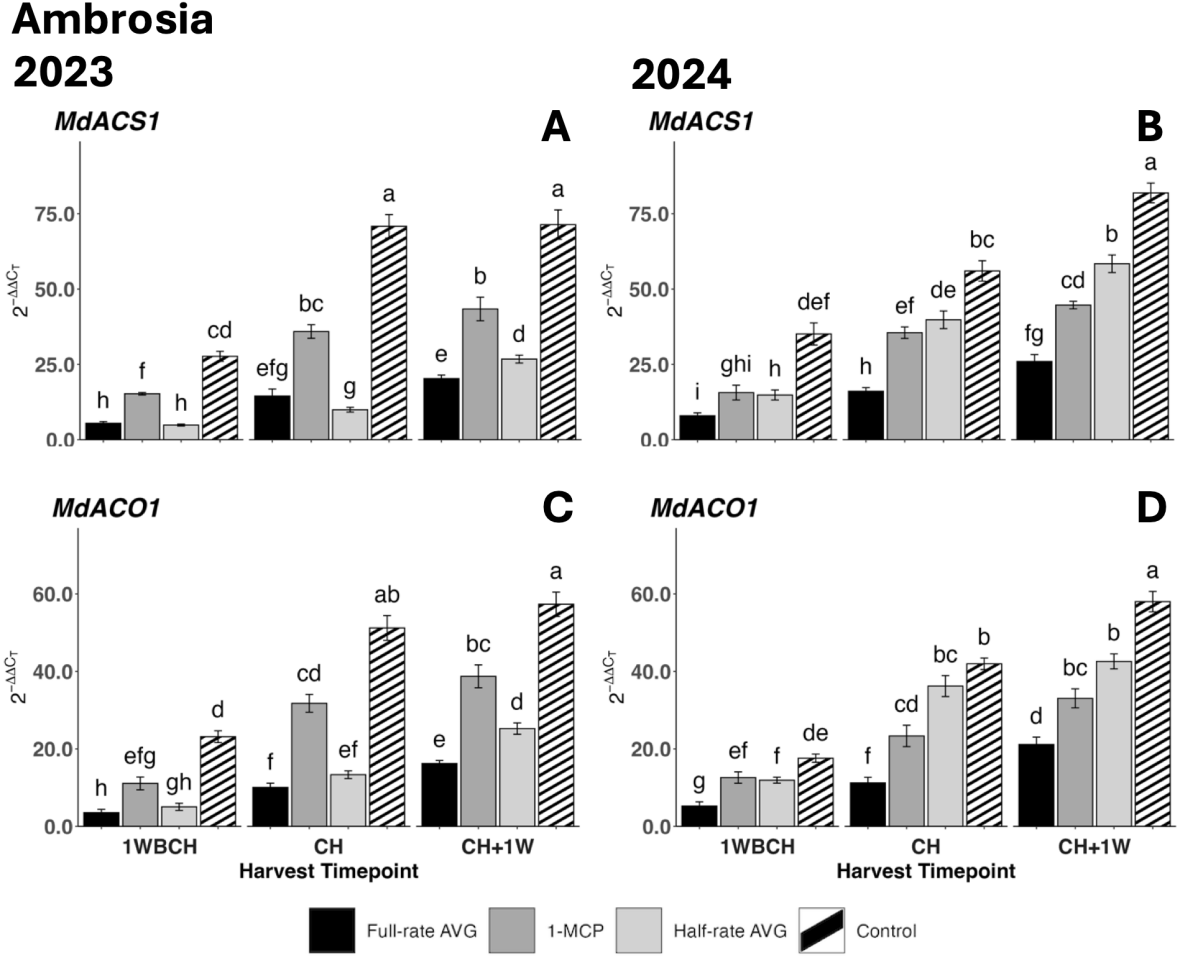
Effect of full-rate AVG, half-rate AVG, and 1-MCP on relative expression of ethylene biosynthesis genes in ‘Ambrosia’ during on-the-tree ripening in Aspers, PA, in **(A, C)** 2023 and **(B, D)** 2024. **(A, B)** *MdACS1* and **(C, D)** *MdACO1*. Apple evaluations were conducted at 1 week before commercial harvest (1WBCH), commercial harvest (CH), and 1 week after commercial harvest (CH + 1W). Values are means ± standard error. Distinct letters represent statistically significant differences (p ≤ 0.05) according to Tukey’s HSD test. ACS, 1-aminocyclopropane-carboxylase synthase; ACO, 1-aminocyclopropane-1-carboxylate oxidase; AVG, aminoethoxyvinylglycine; 1-MCP, 1-methylcyclopropene.

Ethylene biosynthesis genes, *MdACS1* and *MdACO1*, displayed a significant upregulation during on-the-tree ripening from 1WBCH to CH + 1W for all evaluated treatments over both years (**Figures 3A–D**). In 2023, both genes displayed the highest transcript accumulation for control fruits at all stages, followed by 1-MCP-treated fruits, and lastly by half-rate- and full-rate AVG-treated fruits, which exhibited the lowest values (**Figures 3A, C**). The exception to this was at 1WBCH and CH, where full-rate AVG- and half-rate AVG-treated fruits did not differ. The control maintained the highest gene expression levels for both *MdACS1* and *MdACO1*, significantly greater than those of full-rate AVG- and 1-MCP-treated fruits at CH and CH + 1W in 2023 and 2024. 1-MCP-treated fruits followed, displaying intermediate gene expression between both rates of AVG-treated fruits and the control at CH and CH + 1W in 2023. In contrast, in 2024, the expression levels of *MdACS1* in half-rate AVG-treated fruits were greater than those in 1-MCP- and full-rate AVG-treated fruits at CH + 1W (**Figure 3B**).

**Figure 4:**
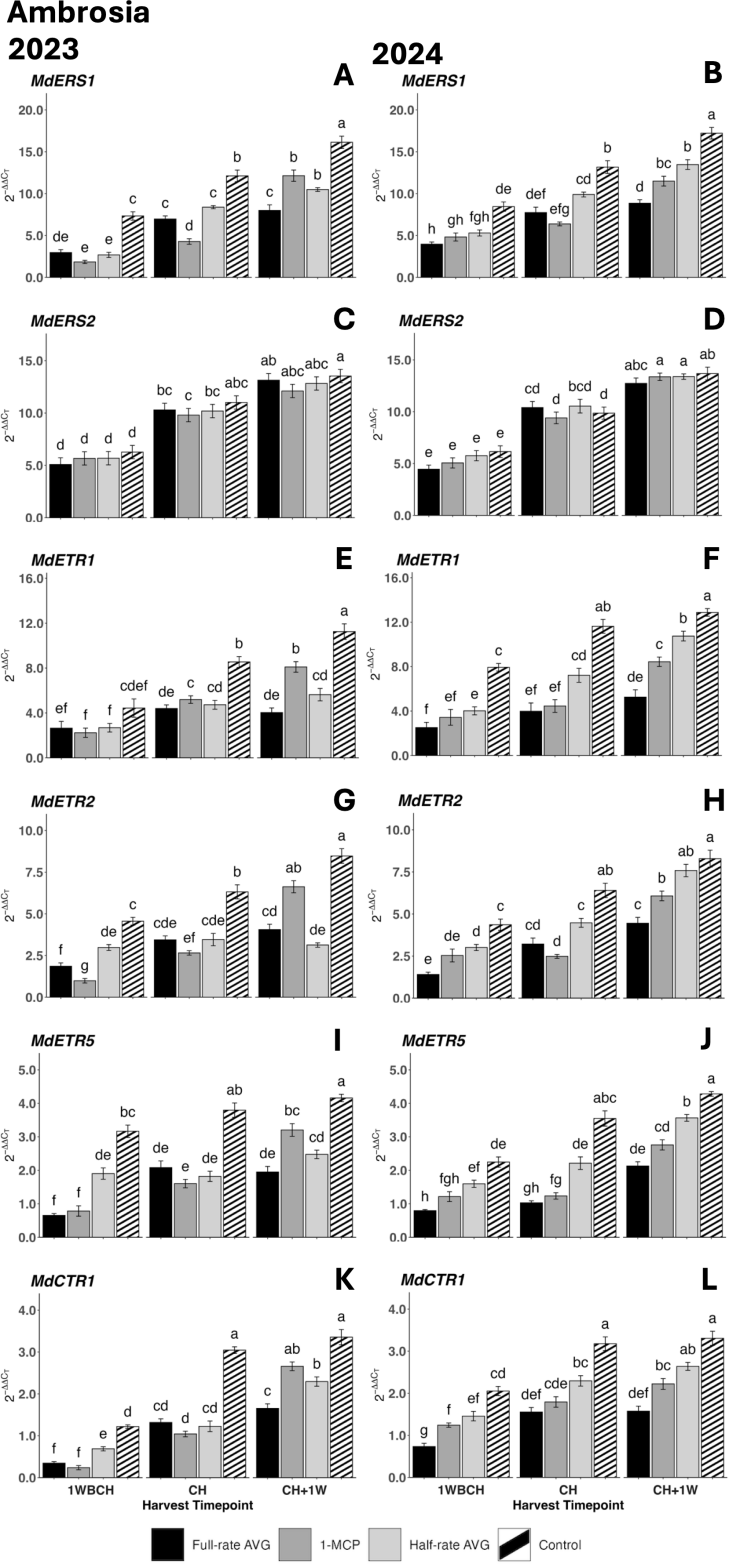
Effect of full-rate AVG, half-rate AVG, and 1-MCP on the relative expression of ethylene perception genes in ‘Ambrosia’ apples during on-the-tree ripening in Aspers, PA, in **(A, C, E, G, I, K)** 2023 and **(B, D, F, H, J, L)** 2024. **(A, B)** *MdERS1*, **(C, D)** *MdERS2*, **(E, F)** *MdETR1*, **(G, H)** *MdETR2*, **(I, J)** *MdETR5*, and **(K, L)** *MdCTR1*. Apple evaluations were conducted at 1 week before commercial harvest (1WBCH), commercial harvest (CH), and 1 week after commercial harvest (CH + 1W). Values are means ± standard error. Distinct letters represent statistically significant differences (p ≤ 0.05) according to Tukey’s HSD test. ERS, ethylene response sensor; ETR, ethylene receptor type; CTR, constitutive triple response; AVG, aminoethoxyvinylglycine; 1-MCP, 1-methylcyclopropene.

Likewise, the expression levels of ethylene perception-related genes rose significantly throughout ripening from 1WBCH to CH + 1W for each treatment (**Figure 4**). The exception to this was *MdETR1*, *MdETR2*, and *MdETR5*, which displayed a significant increase in most but not all treatments from 1WBCH to CH + 1W in 2023 (**Figures 4G, I**). In both years, control fruits displayed significantly higher transcript accumulation than all regulator treatments at CH and CH + 1W for *MdERS1*, *MdETR1*, and *MdETR5*, as well as for *MdCTR1* at CH (**Figures 4A, B, E, F, I–L**). For these same genes at CH + 1W in 2024, the highest expression was observed in half-rate AVG-treated fruits, then 1-MCP-treated fruits, and lastly full-rate AVG-treated fruits. Contrarily, 1-MCP fruits showed significantly higher expression than both AVG treatments at CH + 1W for *MdETR1* and *MdETR2* in 2023 (**Figures 4E, G**). *MdERS2* did not exhibit significant differences among treatments across the harvest timepoints in either year (**Figures 4C, D**).

#### 3.1.5 Expression of anthocyanin biosynthesis genes in ‘Ambrosia’

**Figure 5:**
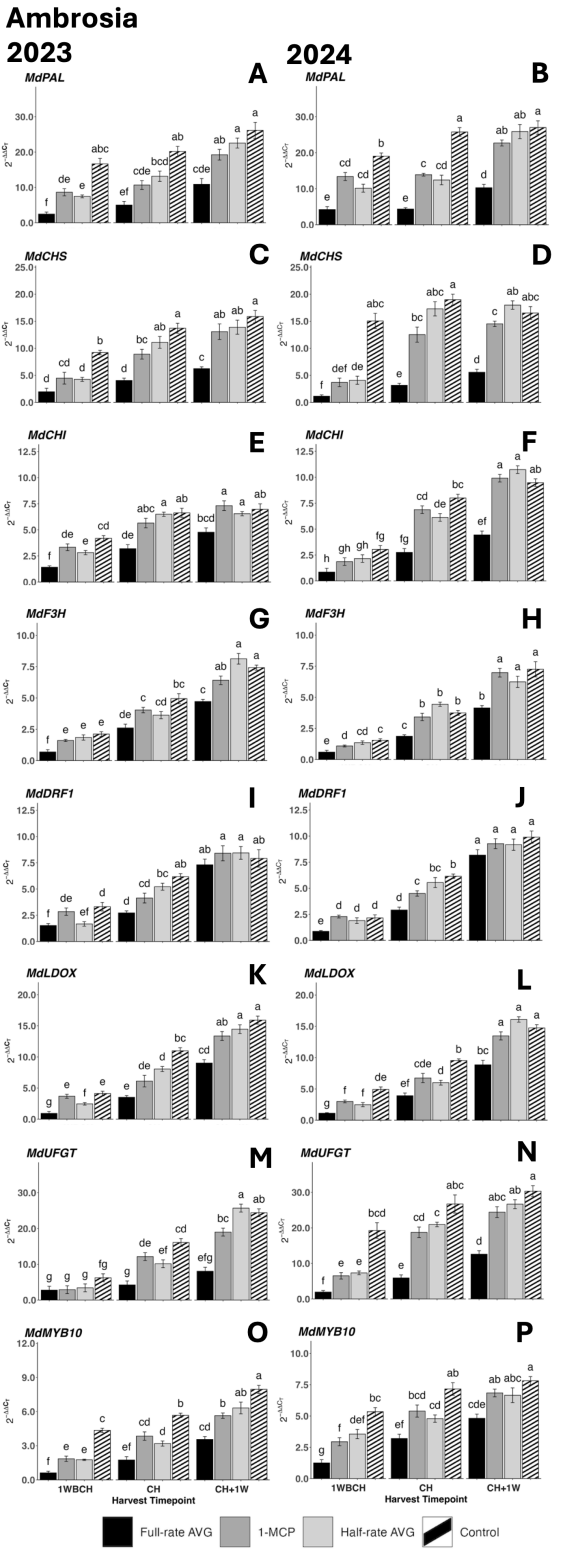
Effect of full-rate AVG, half-rate AVG, and 1-MCP on relative gene expression of anthocyanin biosynthesis-related genes of ‘Ambrosia’ during on-the-tree ripening in Aspers, PA, in **(A, C, E, G, I, K, M, O)** 2023 and **(B, D, F, H, J, L, N,P)** 2024. **(A, B)** *MdPAL*, **(C, D)** *MdCHS*, **(E, F)** *MdCHI*, **(G, H)** *MdF3H*, **(I, J)** *MdDFR1*, **(K, L)** *MdLOX*, **(M, N)** *MdUFGT*, and **(O, P)** *MdMYB10*. Apple evaluations were conducted at 1 week before commercial harvest (1WBCH), commercial harvest (CH), and 1 week after commercial harvest (CH + 1W). Values are means ± standard error. Distinct letters represent statistically significant differences (p ≤ 0.05) according to Tukey’s HSD test. PAL, phenylalanine ammonia-lyase; CHS, chalcone synthase; CHI, chalcone isomerase; F3H, flavanone 3-hydroxylase; DFR, dihydroflavonol 4-reductase; LDOX, leucoanthocyanidin dioxygenase; UFGT, UDP-glucose-flavonoid 3-*O*-glucosyltransferase; MYB, MYB transcription factor; AVG, aminoethoxyvinylglycine; 1-MCP, 1-methylcyclopropene.

Transcript accumulation for all anthocyanin biosynthesis genes increased significantly from 1WBCH to CH + 1W in both years (**Figure 5**). The exception to this was control fruits, which had no significant rise in the expression of *MdPAL* in 2023 or *MdCHS* in 2024, as well as full-rate AVG-treated fruits, which had no significant rise in expression of *MdUFGT* in 2023 (**Figures 5A, D, M**). In both years, AVG-treated fruits displayed the lowest gene expression levels, followed by 1-MCP- and half-rate AVG-treated fruits, and finally by control fruits. The latter presented statistically higher transcript accumulation at CH across all treatments for each gene as compared to full-rate AVG-treated fruits. 1-MCP- and half-rate AVG-treated fruits did not differ at any timepoint for most genes, and 1-MCP-treated, half-rate AVG-treated, and control fruits did not differ at CH + 1W for *MdPAL*, *MdCHI*, *MdF3H*, and *MdLDOX* in both years (**Figures 5A, B, E–H, K, L**). No difference in expression across all treatments was exhibited at CH + 1W for *MdDFR1* in either year (**Figures 5I, J**).

#### 3.1.6 Relationships among fruit drop, cracking, ethylene production, physicochemical parameters, skin color, and gene expression during on-the-tree ripening in ‘Ambrosia’

**Figure 6:**
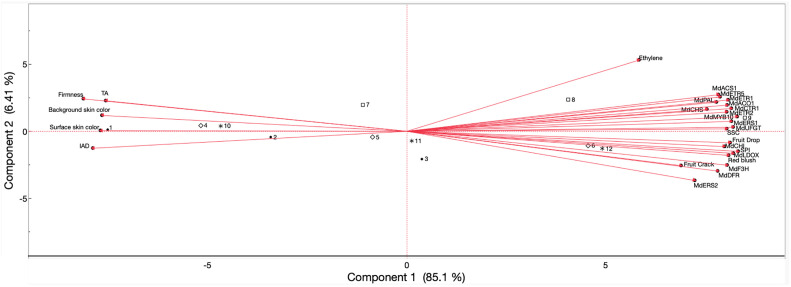
Principal component analysis of values obtained from preharvest fruit drop and cracking, ethylene production, physicochemical properties, skin color, expression of ethylene biosynthesis-related and perception-related genes, and anthocyanin biosynthesis-related genes of ‘Ambrosia’ apples subjected to AVG and 1-MCP treatments and evaluated during on-the-tree ripening. Numbers correspond to the different treatments and evaluation timepoints that were examined. •1, full-rate AVG_1WBCH; •2, full rate-AVG_CH; •3, full-AVG_CH + 1W; ⋄4, 1-MCP_1WBCH; ⋄5, 1-MCP_CH; ⋄6, 1-MCP_CH + 1W; □7, control_1WBCH; □8, control_CH; □9, control_CH + 1W; *10, half-rate AVG_1WBCH; *11, half-rate AVG_CH; *12, half-rate AVG_CH + 1W. SPI, starch pattern index; SSC, soluble solids content; TA, titratable acidity; I_AD,_ index of absorbance difference; AVG, aminoethoxyvinylglycine; 1-MCP, 1-methylcyclopropene. Gene coding is defined in **Figures 3–5**.

Pearson’s correlation coefficients were obtained (**Supplementary Table 2**), and a principal component analysis (**Figure 6**) was performed for ‘Ambrosia’ fruits in 2023 and 2024, including all evaluated parameters in the above results. Preharvest fruit drop and cracking displayed positive correlations with ethylene production rate (r = 0.67 and 0.54, respectively), blush percentage (r = 0.92 and 0.75), SPI (r = 0.95 and 0.85), SSC (r = 0.90 and 0.70), expression of all anthocyanin biosynthesis genes (r ≥ 0.80 and 0.51), and gene expression of ethylene biosynthesis (r ≥ 0.87 and 0.67) and perception (r ≥ 0.83 and 0.68). Drop and cracking were negatively correlated with surface skin hue angle (r = −0.85 and −0.59), background skin hue angle (r = −0.93 and −0.97), I_AD_ (r = −0.86 and −0.64), firmness (r = −0.95 and −0.88), and TA (r = −0.84 and −0.76).

‘Ambrosia’ ethylene production rate displayed positive associations with skin blush percentage (r = 0.49), SPI (r = 0.54), SSC (r = 0.67), expression of all anthocyanin biosynthesis genes (r ≥ 0.42), and gene expression of ethylene biosynthesis (r ≥ 0.78) and perception (r ≥ 0.34). Conversely, ethylene rate exhibited negative correlations with surface skin hue (r = −0.56), background skin hue (r = −0.61), I_AD_ (r = −0.68), firmness (r = −0.49), and TA (r = −0.45).

For physicochemical parameters, firmness presented positive associations with surface skin hue (r = 0.86), background skin hue (r = 0.90), I_AD_ (r = 0.84), and TA (r = 0.93) while showing negative associations with blush percentage (r = −0.96), SPI (r = −0.98), SSC (r = −0.89), expression of all anthocyanin biosynthesis genes (r ≤ −0.80), and gene expression of ethylene biosynthesis (r ≤ −0.79) and perception (r ≤ −0.80). SPI exhibited positive correlations with blush percentage (r = 0.97), SSC (r = 0.92), expression of all anthocyanin biosynthesis genes (r ≥ 0.85), and gene expression of ethylene biosynthesis (r ≥ 0.84) and perception (r ≥ 0.86). Nevertheless, negative correlations were observed between SPI and surface skin hue (r = −0.88), background skin hue (r = −0.90), I_AD_ (r = −0.88), and TA (r = −0.91). The parameter SSC presented positive associations with blush percentage (r = 0.92), expression of all anthocyanin biosynthesis genes (r ≥ 0.82), and gene expression of ethylene biosynthesis (r ≥ 0.91) and perception (r ≥ 0.96) while contrarily displaying significantly negative correlations with surface skin hue (r = −0.89), background skin hue (r = −0.82), I_AD_ (r = −0.88), and TA (r = −0.80). There was a positive association between TA and surface skin hue (r = 0.90), background skin hue (r = 0.77), and I_AD_ (r = 0.84) while correlating negatively with blush percentage (r = −0.94), expression of all anthocyanin biosynthesis genes (r ≤ −0.75), and gene expression of ethylene biosynthesis (r ≤ −0.67) and perception (r ≤ −0.76).

For color-related parameters, surface and background skin hue angle and I_AD_ were positively associated (r ≥ 0.67), yet all exhibited negative associations with blush percentage (r ≤ −0.81), anthocyanin biosynthesis gene expression (r ≤ −0.67), and gene expression of ethylene biosynthesis (r ≤ −0.79) and perception (r ≤ −0.70). Blush percentage displayed juxtaposed positive associations with gene expression of anthocyanin biosynthesis (r ≥ 0.81), ethylene biosynthesis (r ≥ 0.80), and ethylene perception (r ≥ 0.82).

The expression of all anthocyanin biosynthesis-related genes was positively associated with each other (r ≥ 0.73), as was the expression of all ethylene biosynthesis (r ≥ 0.99) and ethylene perception assessed genes (r ≥ 0.66). Additionally, correlations were positive among anthocyanin and ethylene gene expression (r ≥ 0.60) and gene expression of ethylene biosynthesis and perception (r ≥ 0.66).

The PCA showed the allocation of the four treatments across the evaluated harvest timepoints with a total variation of 91.51% explained by the first (85.1%) and second (6.41%) principal components (**Figure 6**). The parameters of TA, firmness, background and surface skin hue angles, and I_AD_ comprised the negative side of the first component axis (associated with full-rate AVG and 1-MCP at 1WBCH and CH, and the control and half-rate AVG at 1WBCH only), while ethylene production, expression of all analyzed anthocyanin and ethylene genes, fruit drop and cracking, blush percentage, SPI, and SSC defined the positive side of the axis (associated with 1-MCP and half-rate AVG at CH + 1W, and the control at CH and CH + 1W). Half-rate AVG at CH and full-rate AVG at CH + 1W were associated with very low positive values of the first component axis.

### 3.2 Experiment 2: effects of AVG and 1-MCP on ‘Fuji’ fruit drop, cracking, ethylene production, physicochemical parameters, skin color, and gene expression during on-the-tree ripening

#### 3.2.1 Fruit drop and cracking in ‘Fuji’

For preharvest fruit drop and cracking, the three treatments (full-rate AVG, 1-MCP, and control) were evaluated throughout on-the-tree ripening at three harvest timepoints (**Table 2**). A rise was observed for preharvest fruit drop from 1WBCH to CH + 1W in 2022, with over an eightfold increase for full-rate AVG and 1-MCP treatments at CH + 1W and over a 20-fold increase for control fruits at CH + 1W, although insignificant.

**Table 2:** Effect of AVG and 1-MCP on ‘Fuji’ preharvest fruit drop and cracking evaluated during on-the-tree ripening in Aspers, PA, during 2022 and 2023 production seasons.

Year	Treatment	Preharvest fruit drop (%)			Preharvest fruit cracking (%)		
		1WBCH	CH	CH + 1W	1WBCH	CH	CH + 1W
2022							
	AVG	0.67 ± 0.38 b	2.16 ± 0.76 ab	5.89 ± 1.18 a	0 ± 0.00 c	0.31 ± 0.31 c	9.43 ± 0.68 a
	1-MCP	0.33 ± 0.33 b	1.29 ± 0.51 b	2.63 ± 1.44 ab	0 ± 0.00 c	0.67 ± 0.38 c	4.92 ± 0.31 b
	Control	0.33 ± 0.33 b	1.34 ± 0.77 ab	7.67 ± 2.63 ab	1.33 ± 0.94 c	2 ± 1.59 bc	10.33 ± 1.00 a
2023							
	AVG	2.95 ± 1.7 a	4.51 ± 1.95 a	7.71 ± 2.15 a	0.61 ± 0.36 b	2.56 ± 0.90 b	15.46 ± 0.94 a
	1-MCP	2.2 ± 0.30 a	5.17 ± 1.81 a	6.05 ± 1.79 a	0.31 ± 0.31 b	3.21 ± 1.11 b	18.36 ± 2.02 a
	Control	1.13 ± 0.48 a	2.32 ± 1.61 a	7.26 ± 3.79 a	0.67 ± 0.67 b	5.34 ± 0.14 b	17.1 ± 1.95 a

Apples were harvested at 1 week before commercial harvest (1WBCH), commercial harvest (CH), and 1 week after CH (CH + 1W). Values are means ± standard error. Distinct letters represent statistically significant differences (p ≤ 0.05) according to Tukey’s HSD test.

AVG, aminoethoxyvinylglycine; 1-MCP, 1-methylcyclopropene.

Regarding fruit cracking, statistically higher values were displayed at CH + 1W compared with 1WBCH for each treatment in 2022 and 2023 (**Table 2**). In 2022, 1-MCP displayed twofold reduction in fruit cracking at CH + 1W compared to control and full-rate AVG-treated fruits.

#### 3.2.2 Ethylene production and physicochemical parameters in ‘Fuji’

**Figure 7:**
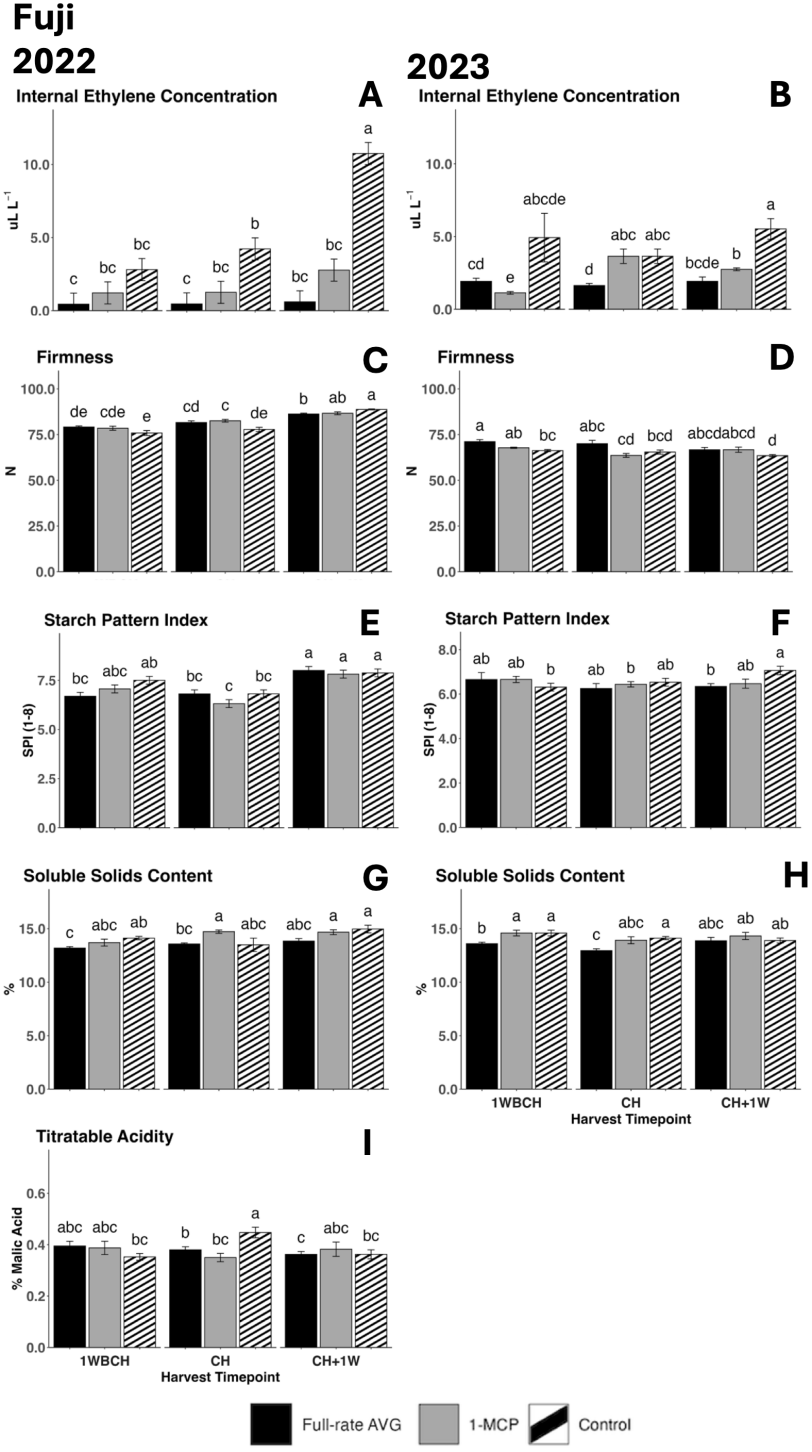
Effect of full-rate AVG and 1-MCP on ‘Fuji’ internal ethylene concentration and physicochemical properties during on-the-tree ripening in Aspers, PA, in **(A, C, E, G, I)** 2022 and **(B, D, F, H)** 2023. **(A, B)** Internal ethylene concentration, **(C, D)** flesh firmness, **(E, F)** starch content (starch pattern index), **(G, H)** soluble solids content, and **(I)** titratable acidity (malic acid). Apple evaluations were conducted at 1 week before commercial harvest (1WBCH), commercial harvest (CH), and 1 week after commercial harvest (CH + 1W). Values are means ± standard error. Distinct letters represent statistically significant differences (p ≤ 0.05) according to Tukey’s HSD test. AVG, aminoethoxyvinylglycine; 1-MCP, 1-methylcyclopropene.

IEC increased significantly for control fruits throughout ripening from 1WBCH to CH + 1W in 2022 (**Figure 7A**) and for 1-MCP-treated fruits in 2023 (**Figure 7B**). At CH, in both years, control fruits only statistically differentiated from full-rate AVG-treated fruits (**Figures 7A, B**), while in 2023, IEC for full-rate AVG-treated fruits was statistically lower than that for 1-MCP-treated fruits (**Figure 7B**). At CH + 1W, in both years, control fruits exhibited significantly higher IEC than the two assessed ethylene-inhibiting regulator treatments (**Figures 7A, B**).

Flesh firmness decreased significantly from 1WBCH to CH + 1W in 2023 in control fruits only (**Figure 7D**). An increased firmness was exhibited in full-rate AVG-treated fruits at 1WBCH as compared to the control in 2023, yet it did not differentiate from 1-MCP-treated fruits or control fruits at any other timepoint. In 2022, 1-MCP-treated fruits displayed significantly higher firmness values compared to the control at CH, while the control was significantly higher than full-rate AVG-treated fruits in firmness at CH + 1W (**Figure 7C**).

SPI values steadily increased during on-the-tree ripening from 1WBCH to CH + 1W for full-rate AVG-treated fruits in 2022, while SPI values for 1-MCP-treated and control fruits remained generally constant (**Figure 7E**). In 2023, only control fruits displayed a significant increase in SPI, as full-rate AVG-treated fruits displayed lower values at CH + 1W compared to control fruits, indicative of increased starch disappearance in the latter (**Figure 7F**).

Regarding SSC, values were constant throughout the three assessed timepoints in all treatments for both years (**Figures 7G, H**). Full-rate AVG-treated fruits exhibited significantly lower SSC as compared to the control at 1WBCH and as compared to 1-MCP-treated fruits at CH in 2022 (**Figure 7G**). In 2023, full-rate AVG-treated fruits exhibited the lowest SSC as compared to all treatments at 1WBCH and presented statistically reduced values than control fruits at CH (**Figure 7H**).

TA generally remained constant across treatments throughout the assessed ripening timepoints in 2022, although control fruits presented significantly higher TA values at CH compared with 1WBCH (**Figure 7I**). Full-rate AVG- and 1-MCP-treated fruits displayed significantly reduced TA values than the control at CH in that same year. TA was not able to be measured during 2023 due to an unexpected technical problem.

#### 3.2.3 Skin coloration in ‘Fuji’

**Figure 8:**
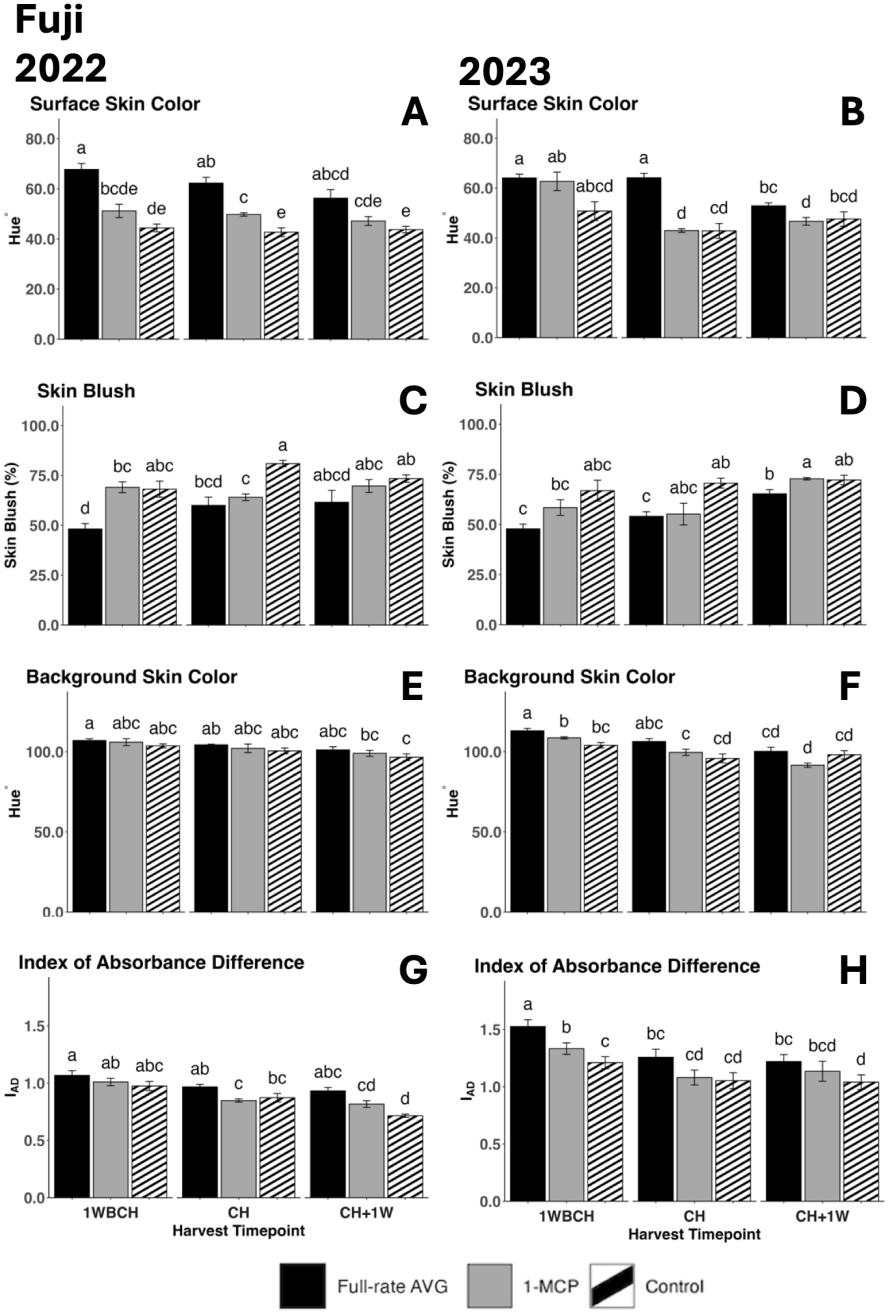
Effect of full-rate AVG and 1-MCP on ‘Fuji’ fruit color during on-the-tree ripening in Aspers, PA, in **(A, C, E, G)** 2022 and **(B, D, F, H)** 2023. **(A, B)** Surface skin color, **(C, D)** skin blush, **(E, F)** background skin color, and **(G, H)** index of absorbance difference. Apple evaluations were conducted at 1 week before commercial harvest (1WBCH), commercial harvest (CH), and 1 week after commercial harvest (CH + 1W). Values are means ± standard error. Distinct letters represent statistically significant differences (p ≤ 0.05) according to Tukey’s HSD test. AVG, aminoethoxyvinylglycine; 1-MCP, 1-methylcyclopropene.

Surface skin hue showed a decreasing trend from 1WBCH to CH + 1W throughout ripening in all treatments in both years (**Figures 8A, B**). In 2022, full-rate AVG-treated fruits showed the highest surface skin values at each timepoint, remaining statistically higher than those of 1-MCP-treated fruits and control fruits at 1WBCH and CH and higher than those of control fruits at CH + 1W (**Figure 8A**). 1-MCP-treated fruits presented statistically increased surface skin values over control fruits at CH (**Figure 8A**). In 2023, full-rate AVG-treated fruits exhibited higher surface skin values than 1-MCP-treated fruits and control fruits at CH and than 1-MCP-treated fruits at CH + 1W (**Figure 8B**). Correspondingly, skin blush percentage presented a tendency to increase as ripening progressed on the tree from 1WBCH to CH + 1W in both years (**Figures 8C, D**). In 2022, full-rate AVG-treated fruits showed significantly decreased blush percentage values than 1-MCP-treated fruits and the control at 1WBCH and than the control at CH (**Figure 8C**). In 2023, full-rate AVG-treated fruits exhibited significantly lower blush than control fruits at CH and 1-MCP-treated fruits at CH + 1W (**Figure 8D**). 1-MCP treatment only significantly reduced blush compared with the control at CH in 2022 (**Figure 8C**). Notably, full-rate AVG was the singular treatment that failed to meet the 50% blush percentage requirement at the first timepoint (1WBCH), only reaching 50% 1 week later at CH in both years.

A significant reduction in background skin hue values throughout the evaluated harvest timepoints from 1WBCH to CH + 1W, revealing a green to yellow color conversion, was observed in both years for most treatments, with significant differences between 1WBCH and CH + 1W for full-rate AVG- and 1-MCP-treated fruits in 2023 (**Figures 8E, F**). Further, full-rate AVG-treated fruits displayed significantly higher background skin values than 1-MCP-treated and control fruits at 1WBCH in 2023 (**Figure 8F**). Correspondingly, I_AD_ values decreased throughout ripening in both years (**Figures 8G, H**). In 2023, at 1WBCH, full-rate AVG treatment produced statistically higher I_AD_ values, followed by 1-MCP, and lastly the control (**Figure 8H**). Full-rate AVG-treated fruits additionally exhibited statistically higher I_AD_ values than control fruits at CH + 1W in both years (**Figures 8G, H**).

#### 3.2.4 Expression of ethylene biosynthesis and perception genes in ‘Fuji’

**Figure 9:**
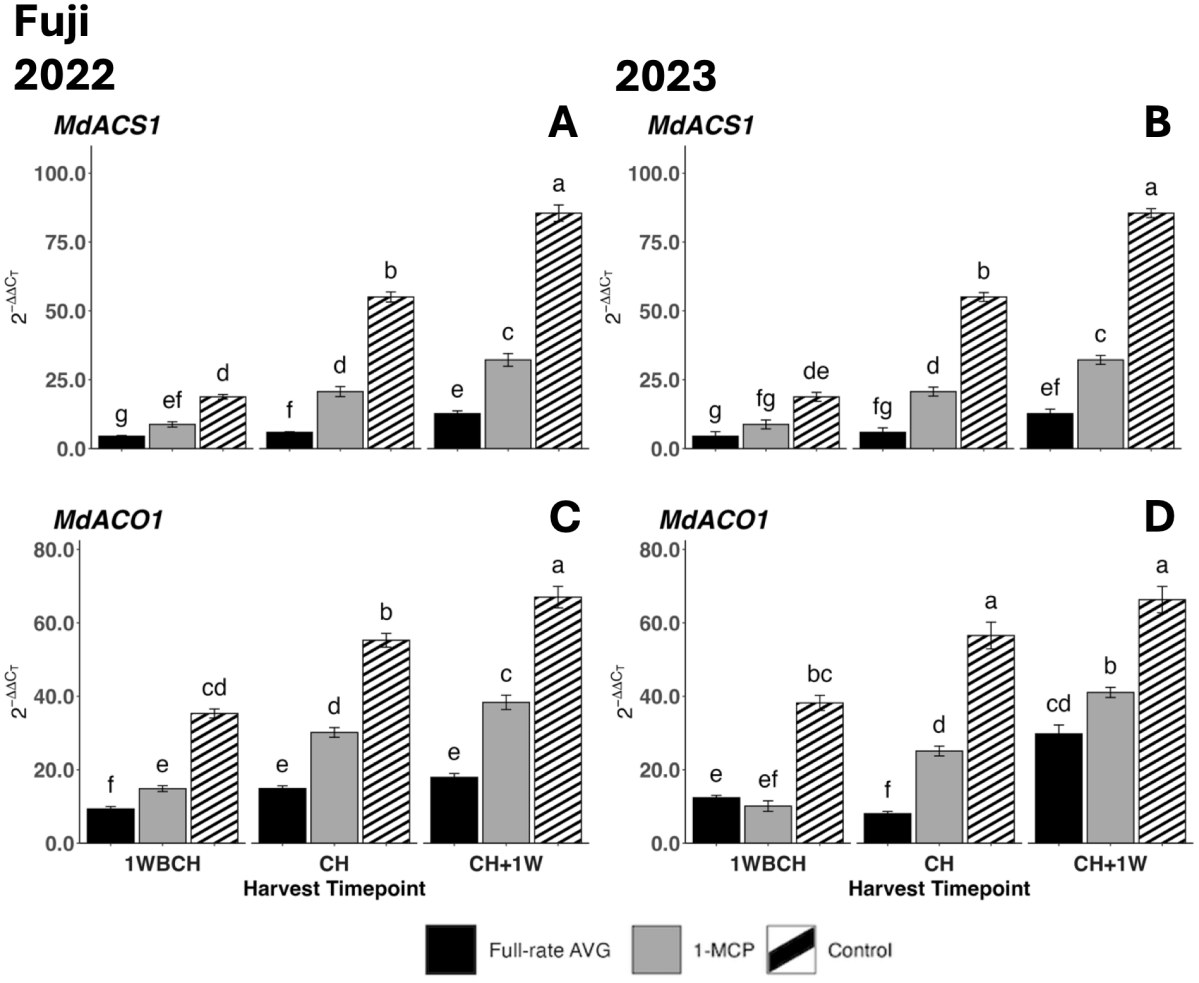
Effect of full-rate AVG and 1-MCP on relative expression of ethylene biosynthesis genes in ‘Fuji’ during on-the-tree ripening in Aspers, PA, in **(A, C)** 2022 and **(B, D)** 2023. **(A, B)** *MdACS1* and **(C, D)** *MdACO1*. Apple evaluations were conducted at 1 week before commercial harvest (1WBCH), commercial harvest (CH), and 1 week after commercial harvest (CH + 1W). Values are means ± standard error. Distinct letters represent statistically significant differences (p ≤ 0.05) according to Tukey’s HSD test. ACS, 1-aminocyclopropane-carboxylase synthase; ACO, 1-aminocyclopropane-1-carboxylate oxidas; AVG, aminoethoxyvinylglycine; 1-MCP, 1-methylcyclopropene.

Transcript accumulation for both assessed ethylene biosynthesis genes, *MdACS1* and *MdACO1*, increased significantly as on-the-tree ripening progressed from 1WBCH to CH + 1W in each treatment in both years (**Figure 9**). In 2022, full-rate AVG-treated fruits displayed the statistically lowest gene expression, followed by 1-MCP-treated fruits, and lastly the control at each timepoint (**Figures 9A, C**). The same trend was observed in 2023 in CH and CH + 1W, while at 1WBCH, there were no differences between full-rate AVG- and 1-MCP-treated fruits (**Figures 9B, D**). Control fruits exhibited significantly higher gene expression values of *MdACS1* and *MdACO1* than either ethylene regulator treatment at all timepoints in both years.

**Figure 10:**
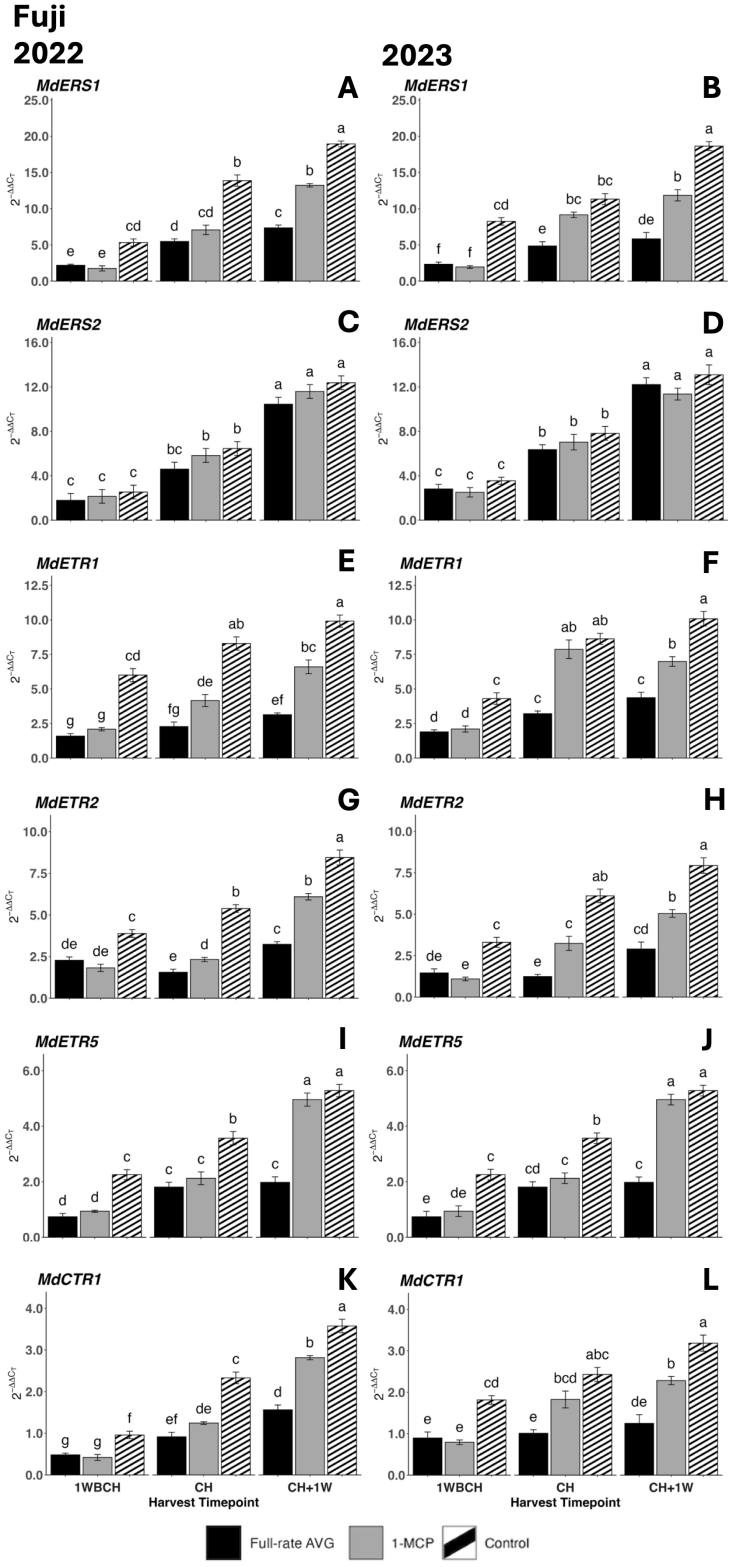
Effect of full-rate AVG and 1-MCP on the relative expression of ethylene perception genes in ‘Fuji’ apples during on-the-tree ripening in Aspers, PA, in **(A, C, E, G, I, K)** 2022 and **(B, D, F, H, J, L)** 2023. **(A, B)** *MdERS1*, **(C, D)** *MdERS2*, **(E, F)** *MdETR1*, **(G, H)** *MdETR2*, **(I, J)** *MdETR5*, and **(K,L)** *MdCTR1*. Apple evaluations were conducted at 1 week before commercial harvest (1WBCH), commercial harvest (CH), and 1 week after commercial harvest (CH + 1W). Values are means ± standard error. Distinct letters represent statistically significant differences (p ≤ 0.05) according to Tukey’s HSD test. ERS, ethylene response sensor; ETR, ethylene receptor type; CTR, constitutive triple response; AVG, aminoethoxyvinylglycine; 1-MCP, 1-methylcyclopropene.

Equally, the transcript accumulation of all ethylene perception genes increased significantly from 1WBCH to CH + 1W in all treatments in both years, except for full-rate AVG treatment for *MdETR2* and *MdCTR1* in 2023 (**Figures 10H, L**). Control fruits displayed significantly higher gene expression values than full-rate AVG- and 1-MCP-treated fruits at 1WBCH for all assessed genes in both years (**Figures 10A, B, E–L)** and at CH in 2022. In 2023, at CH, full-rate AVG-treated fruits exhibited the lowest transcript accumulation values for all assessed genes, while there were no differences between 1-MCP-treated and control fruits for *MdERS1*, *MdETR1*, and *MdCTR1* (**Figures 10B, F, L**). At CH + 1W, for both years, all evaluated genes presented the lowest transcript accumulation for full-rate AVG-treated fruits, followed by 1-MCP-treated fruits, and the statistically highest values were for control fruits, with only *MdETR5* showing no differences between 1-MCP-treated and control fruits. *MdERS2* gene expression levels were an exception to all the above, as treatments exhibiting a lack of significant differences at any timepoint were observed in either production season (**Figures 10C, D**).

#### 3.2.5 Expression of anthocyanin biosynthesis genes in ‘Fuji’

**Figure 11:**
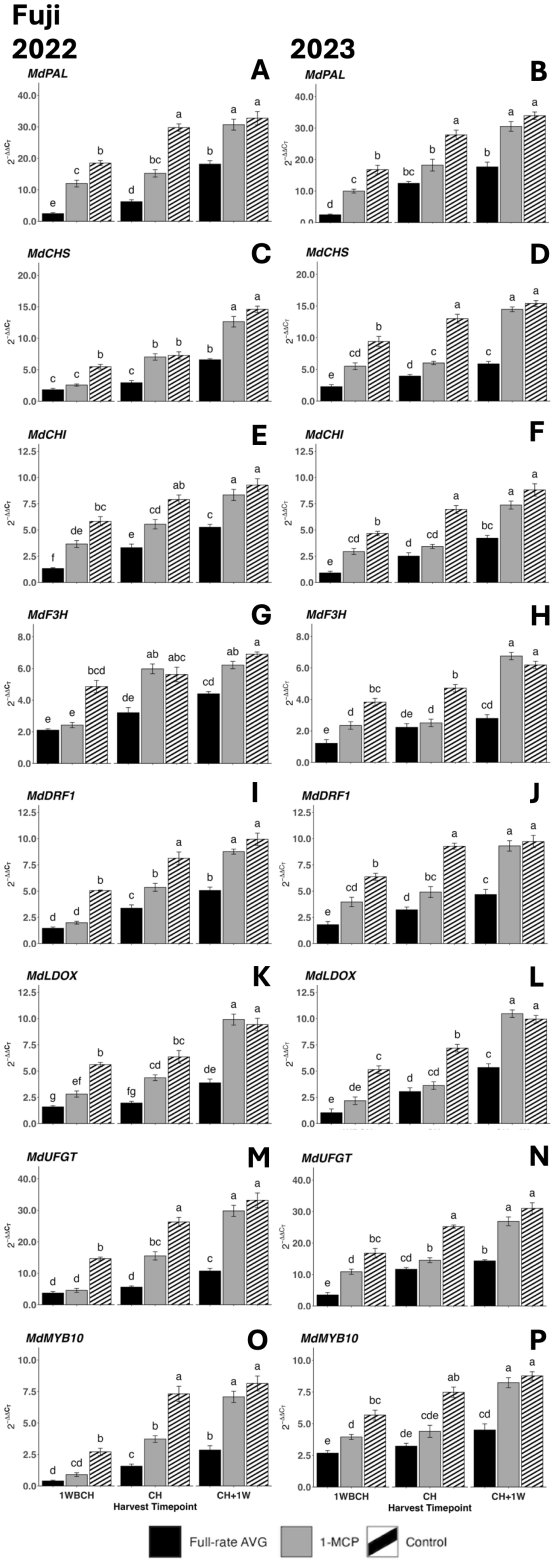
Effect of 1-MCP and full-rate AVG on relative gene expression of anthocyanin biosynthesis-related genes of ‘Fuji’ during on-the-tree ripening in Aspers, PA, in **(A, C, E, G, I, K, M, O)** 2022 and **(B, D, F, H, J, L, N, P)** 2023. **(A, B)** *MdPAL*, **(C, D)** *MdCHS*, **(E, F)** *MdCHI*, **(G, H)** *MdF3H*, **(I, J)** *MdDFR1*, **(K, L)** *MdLOX*, **(M, N)** *MdUFGT*, and **(O, P)** *MdMYB10*. Apple evaluations were conducted at 1 week before commercial harvest (1WBCH), commercial harvest (CH), and 1 week after commercial harvest (CH + 1W). Values are means ± standard error. Distinct letters represent statistically significant differences (p ≤ 0.05) according to Tukey’s HSD test. PAL, phenylalanine ammonia-lyase; CHS, chalcone synthase; CHI, chalcone isomerase; F3H, flavanone 3-hydroxylase; DFR, dihydroflavonol 4-reductase; LDOX, leucoanthocyanidin dioxygenase; UFGT, UDP-glucose-flavonoid 3-*O*-glucosyltransferase; MYB, MYB transcription factor; AVG, aminoethoxyvinylglycine; 1-MCP, 1-methylcyclopropene.

There was a significant rise in gene expression level from the first to last evaluated harvest timepoints for all assessed anthocyanin biosynthesis genes in both years (**Figure 11**). Full-rate AVG-treated fruits presented the statistically lowest transcript accumulation at each timepoint for *MdPAL*, *MdCHI*, and *MdLDOX* in 2022 and for *MdCHS* and *MdDFR1* in 2023, while control fruits displayed the highest transcript accumulation (**Figures 11A, E, K, D, J**). 1-MCP-treated and control fruits presented no significant differences at the final timepoint (CH + 1W) in either year for any gene.

#### 3.2.6 Relationships among fruit drop, cracking, ethylene production, physicochemical parameters, skin color, and gene expression during on-the-tree ripening in ‘Fuji’

**Figure 12:**
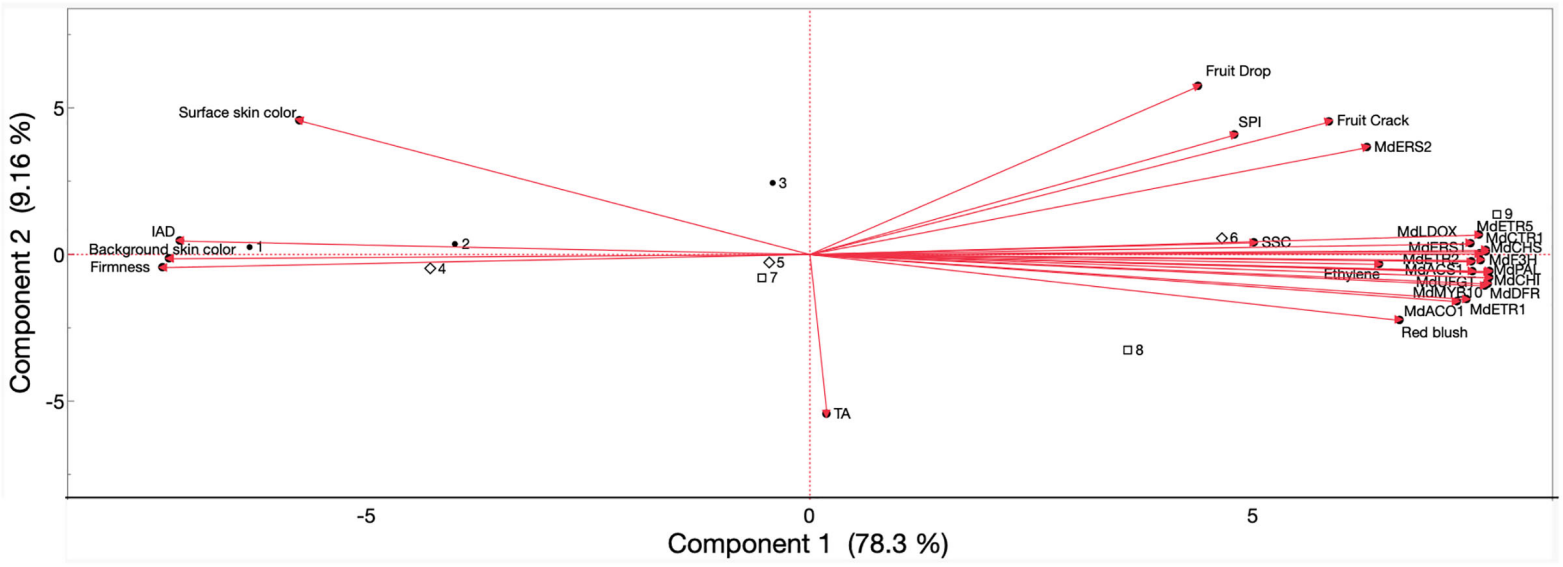
Principal component analysis of values obtained from preharvest fruit drop and cracking, IEC, physicochemical properties, skin color, expression of ethylene biosynthesis-related and perception-related genes, and anthocyanin biosynthesis-related genes of ‘Fuji’ apples subjected to AVG and 1-MCP treatments and evaluated during on-the-tree ripening. Numbers correspond to the different treatments and evaluation timepoints that were examined. •1, full-rate AVG_1WBCH; •2, full-rate AVG_CH; •3, full-rate AVG_CH + 1W; ⋄4, 1-MCP_1WBCH; ⋄5, 1-MCP_CH; ⋄6, 1-MCP_CH + 1W; □7, control_1WBCH; □8, control_CH; □9, control_CH + 1W. SPI, starch pattern index; SSC, soluble solids content; TA, titratable acidity (for 2022 only); I_AD_, index of absorbance difference. Gene coding is defined in **Figures 9–11**. IEC; internal ethylene concentration; AVG, aminoethoxyvinylglycine; 1-MCP, 1-methylcyclopropene.

Pearson’s correlation coefficients were obtained (**Supplementary Table 3**), and a principal component analysis (**Figure 12**) was performed for ‘Fuji’ fruits in 2022 and 2023, including all evaluated parameters in the results above. Preharvest fruit drop and cracking were positively correlated with each another (r = 0.90), and fruit drop correlated less strongly with other parameters over cracking: IEC (r = 0.41 and 0.51, respectively), SPI (r = 0.66 and 0.83), anthocyanin biosynthesis gene expression (r ≥ 0.45 and 0.68), and gene expression of ethylene biosynthesis (r ≥ 0.42 and 0.57) and perception (r ≥ 0.39 and 0.57). Background skin hue angle (r = −0.56 and −0.76), I_AD_ (r = −0.53 and −0.61), and firmness (r = −0.66 and −0.76) all negatively correlated with fruit drop and cracking. Only fruit cracking positively correlated with blush percentage (r = 0.52).

Similarly, IEC rate in ‘Fuji’ fruits displayed positive associations with blush percentage (r = 0.66), SPI (r = 0.57), SSC (r = 0.55), anthocyanin gene expression (r ≥ 0.74), and gene expression of ethylene biosynthesis (r ≥ 0.91) and perception (r ≥ 0.50). Negative associations were exhibited with firmness (r = −0.71), surface skin hue (r = −0.57), background color (r = −0.58), and I_AD_ (r = −0.72).

For the physicochemical parameters, firmness showed positive associations with surface skin hue (r = 0.74), background skin hue (r = 0.95), and I_AD_ (r = 0.96) while presenting negative associations with blush percentage (r = −0.77), SSC (r = −0.57), expression of all anthocyanin genes (r ≤ −0.84), ethylene biosynthesis gene expression (r ≤ −0.86), and ethylene perception gene expression (r ≤ −0.86). SPI, likewise, displayed positive correlations with blush percentage (r = 0.52), SSC (r = 0.48), expression of all anthocyanin genes (r = 0.55), and gene expression of ethylene biosynthesis (r ≥ 0.52) and perception (r ≥ 0.55), except for *MdETR1*. SSC again followed the same positive correlation with blush percentage (r = 0.63), anthocyanin genes (r ≥ 0.60), ethylene biosynthesis (r ≥ 0.56), and perception genes (r ≥ 0.52), with the exception of *MdERS2*, and negative correlations with background skin hue (r = −0.60) and I_AD_ (r = −0.62).

For skin color parameters, blush percentage positively correlated with expression of all anthocyanin genes (r ≥ 0.83), ethylene biosynthesis genes (r ≥ 0.84), and ethylene perception genes (r ≥ 0.75) and negatively correlated with surface skin hue angle (r = −0.85), background skin hue angle (r = −0.81), and I_AD_ (r = −0.79). Conversely, surface skin hue, background skin hue, and I_AD_ were each negatively associated with anthocyanin biosynthesis gene expression (r ≤ −0.65), ethylene biosynthesis gene expression (r ≤ −0.74), and ethylene perception gene expression (r ≤ −0.62), with the exception of *MdERS2*.

Expression of all anthocyanin biosynthesis-related genes showed positive associations with each other (r ≥ 0.96), as did all ethylene biosynthesis (r ≥ 0.97) and ethylene perception assessed genes (r ≥ 0.67). Additionally, correlations were positive among anthocyanin and ethylene gene expression (r ≥ 0.75) and gene expression of ethylene biosynthesis and perception (r ≥ 0.62).

The PCA explained 87.5% of the variation among the three harvest timepoints and the three treatments for all evaluated parameters in the first (78.3%) and second (9.16%) components (**Figure 12**). The negative axis of the first component comprised surface skin color, background skin color, firmness, and I_AD_ and included full-rate AVG at all assessed timepoints (1WBCH, CH, and CH + 1W), 1-MCP at 1WBCH and CH, and the control at 1WBCH. The positive axis comprised parameters preharvest fruit drop and cracking, SPI, SSC, internal ethylene concentration, blush percentage, and all analyzed ethylene- and anthocyanin-related genes and was associated with 1-MCP at CH + 1W and the control at CH and CH + 1W.

## 4 Discussion

Apple fruit yield, marketability, and profitability are directly dependent on preharvest fruit drop, cracking, and physicochemical and color parameters, which can all be altered by the application of ethylene-inhibiting PGRs (Argenta et al., 2022; do Amarante et al., 2022; Liu et al., 2022; Malladi et al., 2023; Johnson and Farcuh, 2024). Ethylene has exhibited a strong influence on preharvest fruit drop and cracking (Klee, 2004; Byers et al., 2005), which is confirmed by the positive associations found between these three parameters in this study. Although not statistically significant potentially due to high data variability, the ~50% reduction in preharvest fruit drop observed in ‘Ambrosia’ fruits with the use of ethylene-inhibiting PGRs compared with control in this work is of critical importance for mid-Atlantic growers, and it also agrees with previous research showing that AVG decreases preharvest fruit drop due to the inhibition of ethylene production in cultivars such as ‘Golden Delicious’ (Sakaldas and Gundogdu, 2016; Byers et al., 2019) and ‘Honeycrisp’ (Arseneault and Cline, 2017; Johnson and Farcuh, 2024), as does 1-MCP in ‘Golden Delicious’ (Sakaldas and Gundogdu, 2016). These trends also align with the reduction in the transcript accumulation of analyzed ethylene biosynthesis and perception genes for both ethylene-inhibiting PGR treatments. Contrarily, the lack of significant reduction in fruit drop with the application of ethylene-inhibiting PGRs in ‘Fuji’ fruits in this study is supported by previous research indicating that this cultivar’s susceptibility to fruit drop has been inconsistent under different environmental conditions where the fruits are grown (Kasai et al., 2008; Li et al., 2010). This study shows that under mid-Atlantic conditions, ‘Fuji’ fruits do not have a high predisposition to fruit drop, which explains the lack of differences among the assessed treatments in this parameter. In fact, AVG drop mitigation has been observed to be most effective when natural drop is heavier (Amarante et al., 2002; Unrath et al., 2023), suggesting that decreased ethylene concentration is not the only factor reducing fruit drop, and weather conditions during the growing season, as well as apple cultivar, play a critical role. Additionally, future studies could consider extending the analyzed time period used in this work to study fruit drop, as it may reveal higher fruit drop percentages and thus significant differences among treatments.

The increase in fruit cracking during on-the-tree ripening for all treatments is consistent with prior findings (Byers, 1997). For ‘Ambrosia’, both ethylene-inhibiting PGR treatments exhibited a reduction in cracking, supported by work in ‘Gala’ (Liu et al., 2022), ‘Arlet’ (Byers et al., 2005), ‘Rome’, and ‘Delicious’ (Byers, 1997). In sweet cherry and litchi fruits, it has been shown that ethylene biosynthesis and fruit cracking share the key genes 1-aminocyclopropane-1-carboxylate synthase (ACS) and ACO (Wang et al., 2019; Michailidis et al., 2021; Santos et al., 2023). The latter could support the alteration of fruit cracking observed in this study after ethylene-inhibiting PGR treatments. Furthermore, higher firmness and delayed fruit ripening may aid in the prevention of fruit cracking (Opara et al., 1997), supporting the impact of ethylene-inhibiting PGRs on decreasing ‘Ambrosia’ fruit cracking in this work. In contrast, the lack of consistent results in ‘Fuji’ for ethylene-inhibiting PGRs reducing fruit cracking differs from earlier studies showing decreased fruit cracking with AVG treatments ranging from 60 to 130 mg a.i. L^−1^ (Petri et al., 2011). These differences could be explained by the major influence on fruit cracking development that location and climate can have, as cracking in ‘Gala’ apples varied considerably across a single orchard (Opara et al., 2000). ‘Fuji’ has been categorized as a cracking-sensitive cultivar, and vulnerability to cracking is additionally advanced by factors like environmental conditions, orchard management, and a lower ability of the fruit’s skin to resist surface tensions due to fruit expansion (Ginzberg and Stern, 2016). In conjunction with the lack of response to ethylene-inhibiting PGR treatment on fruit drop in this study, ‘Fuji’ may require further research into rates and timings of ethylene-inhibiting PGRs to assess the impact on fruit cracking in the mid-Atlantic.

Increased ethylene production throughout ripening in apples is a result of their climacteric nature (Chen et al., 2018; Miah et al., 2023). ACS activity is known to act as the key limiting factor in ethylene biosynthesis during the ripening process, followed by ACO (Brasil and Siddiqui, 2018). The significantly lower ethylene production observed in this work for both cultivars treated with ethylene-inhibiting PGRs, as compared to control fruits, could be a result of ACS enzyme inactivity, which consequently limits ACO. The significantly decreased transcript accumulation of *MdACS1* and *MdACO1* in PGR-treated fruits could be an indirect consequence of this enzyme inactivity and is consistent with prior work in apples (Miah and Farcuh, 2024c) and pears (He et al., 2023). Among ethylene-inhibiting PGR treatments, the most significant impact of full-rate AVG on reducing *MdACS1* and *MdACO1* gene expression levels, compared with 1-MCP, could be explained by its different mode of action. While AVG directly impedes the action of ACS and thus of ethylene biosynthesis, 1-MCP acts as an antagonist to the receptor binding sites of ethylene, binding to and consequently preventing downstream signal transduction (Tatsuki et al., 2007; Bulens et al., 2012), and only then impacting ethylene autocatalysis and reducing the transcript accumulation of ethylene biosynthesis-related genes (Yang and Hoffman, 1984). Research has suggested that 1-MCP also upregulates members of the ERF gene family, which correspond to transcription factors in the last step along the ethylene signaling cascade. ERF members have been shown to act as negative regulators of fruit ripening, ultimately suppressing *MdACS1* transcription (Liu et al., 2015; Li et al., 2016). Furthermore, the lower gene expression levels of *MdACS1* and *MdACO1* in half-rate AVG-treated fruits as compared to full-rate AVG-treated fruits could be a result of a respective reduced degree of ethylene biosynthesis. The latter is consistent with findings in ‘Red Delicious’ exhibiting decreasing ethylene production with increasing AVG rates (Malladi et al., 2023). Moreover, AVG is not an ethylene-specific inhibitor and is known to block other pyridoxal phosphate (PLP)-dependent enzymes in the cell, such as tryptophan aminotransferases (TAAs), which are crucial for auxin biosynthesis (Song et al., 2016). As auxin production appears to trigger ethylene production in apples (Arseneault and Cline, 2016), the application of AVG can potentially suppress the biosynthesis of both hormones, although this needs further study.

Ethylene perception occurs via receptors reported to be negative regulators of downstream ethylene responses (Kevany et al., 2007). As receptor protein degradation is triggered by the binding of ethylene, ethylene binding maintains receptors in the “off” state, and therefore, decreased receptor presence due to degradation results in an enhanced sensitivity to ethylene (Klee and Giovannoni, 2011; Cherian et al., 2014). With the mode of action for 1-MCP directly inhibiting ethylene perception by binding to and preventing the degradation of the receptors, we expected lower expression of perception genes for this treatment with reduced sensitivity to ethylene (Sisler and Serek, 1997). In agreement, in our study, *MdETR1*, *MdETR2*, *MdETR5*, and *MdERS1*, all reported as negative regulators of ethylene in apples and other fruits, such as pear (Xie et al., 2015), displayed significantly lower transcript accumulation for 1-MCP-treated fruits as compared to the control in ‘Fuji’ and ‘Ambrosia’. Full-rate AVG treatment similarly decreased perception-related gene expression, which could be due to the downstream effects of decreased biosynthesis delaying the degradation of the negative ethylene receptors (Kevany et al., 2007; Dal Cin et al., 2008). In fact, AVG treatment at the full rate was generally most effective at reducing the expression of ethylene perception-related genes compared to the other treatments, likely because ethylene biosynthesis was directly impaired by AVG application before perception could occur (Johnson and Farcuh, 2024). Conversely, *MdERS2* presented no significant differences across treatments. This trend in *MdERS2* parallels previous research suggesting that this receptor is ethylene-independent and is instead developmentally dependent, complementary to the rise of expression that we observed throughout the ripening period in both evaluated cultivars (Li and Yuan, 2008; Johnson and Farcuh, 2024). *MdCTR1*, which plays a crucial role as a negative regulator of ethylene, prevents relaying of ethylene signaling and subsequent responses (Kieber et al., 1993; Solano et al., 1998; Alonso et al., 1999; Yang et al., 2013). All ethylene-inhibiting PGR treatments significantly reduced the expression of *MdCTR1* transcripts in this work compared with the control, aligning with prior findings in 1-MCP in ‘Golden Delicious’ (Yang et al., 2013) and both AVG and 1-MCP in ‘Honeycrisp’ (Johnson and Farcuh, 2024). Furthermore, the upregulation of *MdCTR1* expression observed in this work throughout on-the-tree ripening for both cultivars is consistent with previous *CTR1* gene expression studies in different fruits such as plum (Dal Cin et al., 2006; El-Sharkawy et al., 2007; Farcuh et al., 2019), pear (El-Sharkawy et al., 2003), and tomato (Leclercq et al., 2002), suggesting that *MdCTR1*, in its role of negative expression regulator of ethylene responses, may act as a controlling mechanism in response to increased ethylene concentrations, ultimately aiding in slowing the ripening process (Klee, 2002; Yang et al., 2013).

Delayed ethylene production has also been observed to result in delayed fruit ripening on the tree due to the primary role of ethylene in driving apple maturity (Liu et al., 2022). ‘Ambrosia’ fruits treated with ethylene-inhibiting PGRs displayed higher flesh firmness and decreased SPI values (and therefore lower starch disappearance) than control fruits, indicative of delayed maturity. Higher fruit firmness may be a result of the reduced activity of cell wall-degrading enzymes, which have been widely reported to be regulated by ethylene (Johnston et al., 2009; Arseneault and Cline, 2016; Wang et al., 2021; Wu et al., 2023), as observed in other fruits such as melons (Hung et al., 2010) and peaches (Bregoli et al., 2002). Lower starch disappearance parallels prior results in ‘Honeycrisp’ (Johnson and Farcuh, 2024) and ‘Red Delicious’ (Malladi et al., 2023), suggesting that starch degradation may be ethylene-dependent in ‘Ambrosia’. This notion is additionally supported by ethylene production displaying negative associations with firmness and positive associations with SPI in this work. The variable results exhibited by ‘Fuji’ in firmness and SPI with ethylene-inhibiting PGR treatment align with inconsistent values in previous research (Escalada and Archbold, 2019). The decreased SSC values and the higher TA values observed in fruits from both cultivars treated with ethylene-inhibiting PGRs compared with those in the control coincide with prior reports (Argenta et al., 2018a; Tomala et al., 2020a, 2020b; Liu et al., 2022; Doerflinger et al., 2024). The involvement of ethylene in fruit ripening is further supported by ethylene production negatively associating with TA values and positively associating with SSC values in this work, although some studies have attributed a lack of effect on SSC and TA with 1-MCP (McArtney et al., 2008; do Amarante et al., 2022) and AVG (Yuan and Carbaugh, 2007) to seasonal differences, regional location, and application timing (Doerflinger et al., 2019). In general, among ethylene-inhibiting PGR treatments, full-rate AVG was the most efficient in delaying fruit maturity, followed by 1-MCP, and lastly, half-rate AVG. This is likely related to the significantly lower ethylene production in full-rate AVG-treated fruits than in 1-MCP- and half-rate AVG-treated apple fruit. Higher rates of AVG have been documented with greater efficacy than lower rates in delaying apple fruit ripening in prior findings in accordance with higher ethylene biosynthesis inhibition (Greene and Schupp, 2004; Ozturk et al., 2015). The results of the current study are further supported by previous reports indicating that AVG treatment exhibits greater influence on apple fruit maturity than 1-MCP (Scolaro et al., 2015; Argenta et al., 2018b; Johnson and Farcuh, 2024), reinforcing the differential impact of ethylene-inhibiting PGRs on fruit quality.

Red coloration is additionally important for commercial marketability and is the result of anthocyanin accumulation (Whale and Singh, 2007). In our work, ethylene production was positively correlated with the assessed anthocyanin biosynthesis-related genes, suggesting the participation of endogenous ethylene in the regulation of anthocyanin accumulation throughout fruit ripening, in agreement with previous work (Faragher and Brohier, 1984; Blankenship and Richard Unrath, 1988; Wang and Dilley, 2001; Shafiq et al., 2014; Farcuh et al., 2022; Shi et al., 2022). Ethylene production and the assessed anthocyanin biosynthesis genes were moreover positively correlated with blush percentage and negatively correlated with surface skin color, background skin color, and I_AD_. Full-rate AVG in particular has been shown to decrease red coloration in several cultivars, including ‘Gala’ (Argenta et al., 2018b), ‘Cripps Pink’ (Whale et al., 2008), and ‘Honeycrisp’ (Miah and Farcuh, 2024a). Peaches (Belding and Lokaj, 2002) and pears (Clayton et al., 2000) have exhibited similar coloration inhibition. In this study, full-rate AVG-treated fruits reached >50% red blush coverage 1 week later than any other treatment for both assessed cultivars. The latter can be explained by the patterns observed in the analyzed anthocyanin biosynthesis-related genes, where the lowest transcript accumulation was observed in full-rate AVG treatment. Half-rate AVG- and 1-MCP-treated fruits displayed intermediate reductions in coloration and color-related transcript accumulation compared with control and full-rate AVG-treated fruits, which could be due to the different modes of action of the ethylene-inhibiting PGRs and the different rates of AVG. Transcription factor *MdMYB10* plays a key role in regulating anthocyanin biosynthesis and has been shown to positively interact with ethylene biosynthesis genes ACS and ACO (Wang and Dilley, 2001; Espley et al., 2007; Jia et al., 2020; Li et al., 2022; Shi et al., 2022). The differences in red skin coloration among ethylene-inhibiting PGR treatments could also be explained by the fact that AVG directly inhibits ACS and therefore may alter *MdMYB10* transcript accumulation and thus red color development beyond 1-MCP treatment, as 1-MCP represses ethylene perception (Byers, 1997; Yuan and Li, 2008).

The differences discussed above were summarized in the PCA for both ‘Fuji’ and ‘Ambrosia’, where the positioning of full-rate AVG-treated fruits can be explained by these fruits presenting the lowest ethylene production, ethylene biosynthesis and perception, and anthocyanin biosynthesis-related gene expression, and thus the most delayed fruit maturity. A midway maturity relative to full-rate AVG-treated and control fruits for all assessed features was associated with 1-MCP- and half-rate AVG-treated fruits, while the most advanced maturity was exhibited by control fruits. Regarding color, the lowest values for apple skin blush and anthocyanin-related gene expression in full-rate AVG-treated fruits contribute to their placement in the PCA and explain why these fruits only met the minimum requirement of 50% red blush coverage 1 week later than all other treatments in both assessed cultivars. Current work is ongoing in assessing the impact of 1-MCP and AVG on these economically important apple cultivars during postharvest storage.

Our study on the impact of different ethylene-inhibiting plant growth regulators on emerging and prevalent cultivars such as ‘Ambrosia’ and ‘Fuji’ throughout on-the-tree ripening in the mid-Atlantic showed that ethylene production was strongly correlated with fruit drop and that full-rate AVG treatment most efficiently decreased ethylene production as compared to other treatments. Moreover, this work revealed a reduction in fruit cracking compared with control fruits throughout ripening with full-rate AVG and 1-MCP treatments. Full-rate AVG further downregulated the transcript accumulation of ethylene biosynthesis- and perception-related genes, ultimately resulting in the greatest delay to fruit ripening relative to the other treatments in this study. Nevertheless, full-rate AVG treatment presented a challenge for red skin color development, reaching the 50% blush only 1 week after all other treatments trialed in this study in both assessed cultivars. Furthermore, this work demonstrated that although 1-MCP- and half-rate AVG-treated fruits did not delay red skin color development to the extent of the full-rate AVG treatment, they exhibited a midway maturity between the latter and control fruits, revealed by their intermediate ethylene production rate and related gene expression values. This study provides a promising framework for mid-Atlantic growers to select the best ethylene-inhibiting plant growth regulator to aid in harvest and fruit quality management in these economically important apple cultivars.

## Data Availability

The original contributions presented in the study are included in the article/**Supplementary Material**. Further inquiries can be directed to the corresponding author.
