# Supplementary material for: Assessing the impacts of aminoethoxyvinylglycine and 1-methylcyclopropene on fruit drop, cracking, quality, and related transcript accumulation in ‘Ambrosia’ and ‘Fuji’ apples during on-the-tree ripening
Source: Front Plant Sci. 2025 Aug 22;16:1629445. doi: 10.3389/fpls.2025.1629445 (PMC12411216; doi:10.3389/fpls.2025.1629445)
Supplement: Supplementary file 1 [file Table1.docx]

Supplementary Material

**Supplementary Table S1.** Primers used in qRT-PCR.

| **Gene name** | **Description** | **Primer orientation** | **Primer sequence**  **(5’ to 3’)** |
| --- | --- | --- | --- |
| *MdPAL* | Phenylalanine ammonia-lyase | Forward | GTGCTGTGGAGTCCCCGCTT |
|  |  | Reverse | GGTGA GGCTCTCTCCGCCAAGT |
| *MdCHS* | Chalcone synthase | Forward | GGAGACAACTGGAGAAGGACTGGAA |
|  |  | Reverse | CGACATTGATACTGGTGTCTTCA |
| *MdCHI* | Chalcone isomerase | Forward | GGGATAACCTCGCGGCCAAA |
|  |  | Reverse | GCATCCATGCCGGAAGCTACAA |
| *MdF3H* | Flavanone 3-hydroxylase | Forward | TGGAAGCTTGTGAGGACTGGGGT |
|  |  | Reverse | CTCCTCCGATGGCAAATCAAAGA |
| *MdDFR* | Dihydroflavonol 4-reductase | Forward | GATAGGGTTTGAGTTCAAGTA |
|  |  | Reverse | TCTCCTCAGCAGCCTCAGTTTTCT |
| *MdLDOX* | Leucoanthocyanidin dioxygenase | Forward | CCAAGTGAAGCGGGTTGTGCT |
|  |  | Reverse | CAAAGCAGGCGGACAGGAGTAGC |
| *MdUFGT* | UDP glucose-flavonoid 3- o -glucosyl transferase | Forward | CCACCGCCCTTCCAAACACTCT |
|  |  | Reverse | ACCATGTCGTCGTTGGAGTAG |
| *MdMYB10* | Transcription factor | Forward | TGCCTGGACTCGAGAGGAAGACA |
|  |  | Reverse | CCTGTTTCCCAAAAGCCTGTGAA |
| *MdACS1* | 1-aminocyclopropane-carboxylase (ACC) synthase | Forward | CTCCTCCTTTCCTTCGTTGA |
|  |  | Reverse | ACCATGTCGTCGTTGGAGTAG |
| *MdACO1* | ACC oxidase | Forward | ATCAATGATGCTTGTGAGAACTG |
|  |  | Reverse | GGTCTTCTTGTAGTGATCCTTGG |
| *MdERS1* | Ethylene-response sensor | Forward | TCCAGAACTGGTATGAACCTACA |
|  |  | Reverse | AGAACTGTTGAAGACTTCGTTGA |
| *MdERS2* | Ethylene-response sensor | Forward | TGCGAAACCAGAATCTTCAAGA |
|  |  | Reverse | CCTCAGTTGACGCTGGATAAAA |
| *MdETR1* | Ethylene receptor-type | Forward | GCACCTAGGATGTGATGTAACAG |
|  |  | Reverse | TCATGTATACGGACAGCAAGTTC |
| *MdETR2* | Ethylene receptor-type | Forward | AGGCAAACAAAGGGATGACA |
|  |  | Reverse | AGAGTTAATTTCATGCCCGTATAA |
| *MdETR5* | Ethylene receptor-type | Forward | GTTCTTCCGGTTGCAGATTC |
|  |  | Reverse | ATGCATTGGCCTTCTCATTC |
| *MdCTR1* | Constitutive triple response | Forward | ACAAGATTTTCATGCCGAAC |
|  |  | Reverse | TATGGACAAGTTTGGAGGCT |
| *MdACT* | Actin | Forward | TGACCGAATGAGCAAGGAAATTACT |
|  |  | Reverse | TACTCAGCTTTGGCAATCCACATC |
